# Genome, host genome integration, and gene expression in *Diadegma fenestrale* ichnovirus from the perspective of coevolutionary hosts

**DOI:** 10.3389/fmicb.2023.1035669

**Published:** 2023-02-17

**Authors:** Juil Kim, Md-Mafizur Rahman, A-Young Kim, Srinivasan Ramasamy, Min Kwon, Yonggyun Kim

**Affiliations:** ^1^Agriculture and Life Science Research Institute, Kangwon National University, Chuncheon, Republic of Korea; ^2^Program of Applied Biology, Division of Bio-Resource Sciences, College of Agriculture and Life Science, Kangwon National University, Chuncheon, Republic of Korea; ^3^Department Biotechnology and Genetic Engineering, Faculty of Biological Science, Islamic University, Kushtia, Bangladesh; ^4^Ilsong Institute of Life Science, Hallym University, Seoul, Republic of Korea; ^5^World Vegetable Center, Tainan, Taiwan; ^6^Research Institute of Agriculture and Life Sciences, Seoul National University, Seoul, Republic of Korea; ^7^Department of Plant Medicals, College of Life Sciences, Andong National University, Andong, Republic of Korea

**Keywords:** polydnavirus, koinobiont, Campopleginae, coevolution, host adaptation, DfIV, Saesbyeol virus

## Abstract

Polydnaviruses (PDVs) exhibit species-specific mutualistic relationships with endoparasitoid wasps. PDVs can be categorized into bracoviruses and ichnoviruses, which have independent evolutionary origins. In our previous study, we identified an ichnovirus of the endoparasitoid *Diadegma fenestrale* and named it DfIV. Here, DfIV virions from the ovarian calyx of gravid female wasps were characterized. DfIV virion particles were ellipsoidal (246.5 nm × 109.0 nm) with a double-layered envelope. Next-generation sequencing of the DfIV genome revealed 62 non-overlapping circular DNA segments (A1–A5, B1–B9, C1–C15, D1–D23, E1–E7, and F1–F3); the aggregate genome size was approximately 240 kb, and the GC content (43%) was similar to that of other IVs (41%–43%). A total of 123 open reading frames were predicted and included typical IV gene families such as repeat element protein (41 members), cysteine motif (10 members), vankyrin (9 members), polar residue-rich protein (7 members), vinnexin (6 members), and N gene (3 members). Neuromodulin N (2 members) was found to be unique to DfIV, along with 45 hypothetical genes. Among the 62 segments, 54 showed high (76%–98%) sequence similarities to the genome of *Diadegma semiclausum* ichnovirus (DsIV). Three segments, namely, D22, E3, and F2, contained lepidopteran host genome integration motifs with homologous regions of about 36–46 bp between them (*Diadegma fenestrale* ichnovirus, DfIV and lepidopteran host, *Plutella xylostella*). Most of the DfIV genes were expressed in the hymenopteran host and some in the lepidopteran host (*P. xylostella*), parasitized by *D. fenestrale*. Five segments (A4, C3, C15, D5, and E4) were differentially expressed at different developmental stages of the parasitized *P. xylostella*, and two segments (C15 and D14) were highly expressed in the ovaries of *D. fenestrale*. Comparative analysis between DfIV and DsIV revealed that the genomes differed in the number of segments, composition of sequences, and internal sequence homologies.

## 1. Introduction

Parasitoid wasps of the family Ichneumonidae represent a diverse, species-rich group of insects (Gómez et al., 2018) that can regulate their hosts to ensure successful parasitism (Stoltz and Vinson, 1979). The genus *Diadegma* represents a large group of parasitoid wasps with 409 known species worldwide (Sharkey et al., 2013; Klopfstein et al., 2019), including 55 species within 13 genera in the Korean peninsula (Choi and Lee, 2018). In Korea, two koinobiont endoparasitoids of the diamondback moth, *Plutella xylostella*, exist: *D. semiclausum* (Hellen) and *D. fenestrale* (Holmgren; Hymenoptera: Ichneumonidae; Nam et al., 2022). *Diadegma semiclausum* is thought to have originated in Eurasia (Fitton, 1992) and is a specific endoparasitoid of *P. xylostella* larvae. The generalist *D. fenestrale* is polyphagous in nature and attacks over 90 herbivore species (Thompson, 1951; Azidah et al., 2000), including *P. xylostella* and the potato tuber moth, *Phthorimaea operculella*.

During oviposition, parasitoid wasps lay eggs containing a parasitoid-associated polydnavirus (PDV; Tanaka et al., 2007; Dicke et al., 2020). PDVs belong to the family Polydnaviridae, which comprises two genera, namely, *Bracovirus* and *Ichnovirus* (Stoltz and Vinson, 1979; Federici and Bigot, 2003; Lorenzi et al., 2022). The PDV genome comprises encapsidated double-stranded DNA and is 187–567 kb in length (Dupuy et al., 2006; Webb et al., 2006; Tanaka et al., 2007; Wang et al., 2021). PDV particles exist in the parasitoid wasp host ovaries, particularly in the calyx tissues located at the base of the oviducts, where they assemble with the eggs (Stoltz and Vinson, 1979). PDV virions are then injected into hosts during oviposition and can replicate alone or in conjunction with other factors (i.e., virulence factors and integration of genes into the wasp genome) that actively suppress host immunity, promoting the survival of the parasitoids (Edson et al., 1981; Luckhart and Webb, 1996; Moreau et al., 2009; Kashkouli et al., 2021). The host specificity of PDVs depends on their evolutionary lineage (Stoltz and Vinson, 1979). Several PDV virulence genes have seemed to be transcribed in hosts, possibly providing functions on PDV role in parasitism (Strand and Burke, 2013). PDV parasitism rates vary depending on the host, with *Diadegma fenestrale* ichnovirus (DfIV) showing a different parasitism rate in *P. xylostella* and *P. operculella* (Kim et al., 2015).

*Campoletis sonorensis* ichnovirus (CsIV) was the first identified PDV (Norton et al., 1975). To date, eight encapsidated IV genomes, including the Campoplegine and Banchine IV groups, have been fully sequenced and appear to share the canonical ichnovirus gene families (Webb et al., 2006; Lapointe et al., 2007; Tanaka et al., 2007; Djoumad et al., 2013; Doremus et al., 2014; Wang et al., 2021). These two IV groups differ morphologically, with campoplegine IVs possessing singly enveloped nucleocapsids and banchine IVs possessing several smaller nucleocapsids. Banchine IV genome, including Glypta fumiferanae ichnovirus (GfIV) and *Apophua simplicipes* ichnovirus (AsIV) genome, shares an approximately 260-bp region sequence not found in campoplegine IV circles (Djoumad et al., 2013). The function of this region is currently unknown, but it is thought to be involved in the integration of GfIV genome segments into the genome of the lepidopteran host, similar to how host integration motifs in *D. semiclausum* Ichnovirus genome segments (Wang et al., 2021). In addition, eight encapsidated genomes have similar genome sizes (~250 kb) except *Diadegma semiclausum* ichnovirus (DsIV, 208 kb), with the number of DNA segments ranging from 20 (*Tranosema rostrale* ichnovirus, TrIV) to 62 (DfIV). DfIV and DsIV exhibited all the known major gene groups representative of ichnoviruses, and their overall gene counts were similar to those of HfIV and CsIV (Kim et al., 2015; Nam et al., 2022). Nevertheless, the individual T*ranosema rostrale* (TrIV) and *Hyposoter fugitivus* (HfIV) ichnoviruses have special features, including similar genome lengths (∼250 kb), nested genome segments, and members of six conserved gene families (Tanaka et al., 2007). In addition, TrIV has a distinct gene family and more open reading frames (ORFs) than HfIV. The interspecific variations could be related to differences in parasitoid host biology (Tanaka et al., 2007).

DsIV was identified in *D. semiclausum,* and its viral gene contents were partially assessed from the parasitized host, *P. xylostella* (Wang et al., 2021), but the expression of PDV genes in parasitized hosts remains poorly explored (Etebari et al., 2011). The gene expression pattern of DfIV varies across hosts, with higher expression in *P. operculella* than in *P. xylostella* (Kim et al., 2015). However, the factors determining host range (i.e., lepidopteran host) and preference in IVs remain to be understood. Previous studies have suggested that the virus uses virulence factors to manipulate host immune systems to enable the survival of parasitoid eggs and larvae (Herniou et al., 2013; Kashkouli et al., 2021). PDVs contain functional genes that assist in manipulating host physiological functions, for example through transcription inhibition (Shelby et al., 1998; Barandoc and Kim, 2009). Several research groups have attempted to investigate the genomic composition of PDVs to isolate functional genes, and genomic characterization has been attempted to gain insight into the mechanisms of action of these viruses (Barat-Houari et al., 2006; Lapointe et al., 2007; Tanaka et al., 2007; Choi et al., 2009). However, few genes have been identified to characterize those that may be involved in physiological functions.

Although DfIV was identified before DsIV and DfIV gene expression has been studied (Kim et al., 2015), its genome has not yet been identified. In this study, we attempted to elucidate the relationship between parasitoid organisms and PDV from an evolutionary point of view by sequencing the DfIV genome and comparing DfIV to those of symbiotic DsIV genome, DsIV from *D. semiclausum*, which have very closely related evolutionary lineages. Furthermore, we compared PDV gene expression in two major PDV hosts, the lepidopteran moth *P. xylostella*, and the parasitoid wasp *D. fenestrale*. To confirm the presence of DfIV in the ovary, microscopic analysis was performed by using transmission electron microscopy (TEM). We identified three integration motifs in lepidopteran hosts, as well as a novel gene family, hypothetical genes, their putative functions, and gene expression patterns in respective hosts. Our findings highlight new genes and elucidate their interactions, integration, and expression, improving our understanding of the coevolution of hosts and viruses.

## 2. Materials and methods

### 2.1. Insects

*Diadegma fenestrale* was obtained by infesting potato fields with the parasitized larval stage of *Phthorimaea operculella* (Zeller), in Jeju, Korea, in May 2009. Specimens were collected and maintained in the laboratory in Pyeongchang, Korea (Choi et al., 2013). *Diadegma semiclausum* was donated by the World Vegetable Center (former Asian Vegetable Research and Development Center, AVRDC), Taiwan, in 2001 (Kwon et al., 2003; Nam et al., 2022). *Diadegma fenestrale* was reared on *P. operculella* or *P. xylostella* as hosts in transparent plastic cages (30 × 30 × 30 cm) under the conditions of 25°C ± 2°C, 16:8-h light:dark photoperiod, and 50%–70% relative humidity. Third instar *P. operculella* or *P. xylostella* larvae (5 days after hatch) were parasitized by *D. fenestrale* in an open-type cylindrical plastic cage (15 cm diameter, 30 cm height) for 24 h, and parasitized hosts were reared in conditions similar to those used for unparasitized hosts. The emerged *D. fenestrale* adults were collected every day and allowed to mate for 24 h before use for parasitization. Adult wasps were fed with a 10% sucrose solution. Rearing methods and conditions for *D. semiclausum* were similar to those used for *D. fenestrale,* but *P. xylostella* was used as a moth host.

### 2.2. Genomic DNA extraction from DfIV

*Diadegma fenestrale* ovaries from 1-day-old female wasps were dissected, and tissues from more than 100 female parasitoids were collected in phosphate-buffered saline (PBS). The dissected ovary tissues were homogenized using a glass–glass micro-tissue grinder (Radnoti, Monrovia, CA, United States), and the homogenate was passed through a 0.45 μm syringe filter (Advantec MFS Inc., Dublin, CA, United States) and centrifuged for 30 min at 15,000 ×*g* and 4°C. The pellet was resuspended in DNAzol (Molecular Research Center Inc., Cincinnati, Ohio, United States) and homogenized using a disposable tissue grinder. The resulting homogenate was centrifuged for 15 min at 12,000 ×*g* and 4°C, and then, the supernatant was transferred to a new tube. The DfIV genomic DNA (gDNA) was precipitated by adding the same volume of ethanol and centrifuging for 10 min at 10,000 ×*g* and 4°C. The pellet was washed with 75% ethanol, dried, and then resuspended in nuclease-free water. DfIV gDNA was quantified using a NanoDrop ND-100 spectrophotometer (NanoDrop Technologies, Wilmington, DE, United States). To visualize the viral segment DNAs, 2 μg of DfIV gDNA was separated on 0.5% agarose gel at 30 V for 9 h. The gDNA of DsIV was isolated following the methods used for DfIV.

### 2.3. DfIV and DsIV genome sequencing and annotation

To characterize the genome composition of DfIV viral particles, extracted gDNA was subjected to 454 pyrosequencing (Science, 2008). Whole-genome shotgun sequencing of DfIV was performed at Macrogen Inc. (Seoul, Korea) using the Roche 454 GS-FLX sequencer (Roche, Basel, Switzerland) with FLX-plus chemistry sets according to the GS-FLX manual. After shotgun sequencing, the adapter and primer sequences were removed, and the DfIV genome was assembled using the Newbler ver. 2.6 GS *de novo* assembler (Roche). To improve the genome sequences, resequencing of the DfIV genome was performed through Illumina paired-end library construction and MiSeq platform sequencing (Illumina, San Diego, CA, United States). The sequencing data were trimmed to remove the adapter and low-quality reads (Q20, 1-bp error read in each 100-bp sequencing; call accuracy of 99% and Q30% indicates virtual reads will be perfect with no ambiguities) and then assembled using SOAPdenovo ver. 2.0 (Xie et al., 2014) and DNASTAR Lasergene ver. 14.1.0 (DNASTAR, Inc., Madison, WI, United States) with default parameters. Contigs with similarity to the DsIV genome were selected based on BLAST (Zhang et al., 2000) searches against the non-redundant nucleotide sequence in GenBank. The DfIV genome was annotated using BLAST against *Diadegma semiclausum* ichnovirus sequences,^1^ *ab initio* gene prediction in GeneMark (Besemer and Borodovsky, 2005) and FGENESV0 (Solovyev et al., 2006), and final manual curation. The DsIV genome was sequenced through the same methods using the Illumina platform. Sequence similarity between DfIV and DsIV genome segments was analyzed using reciprocal BLAST searches and visualized using Circos (http://circos.ca/; Krzywinski et al., 2009). Open reading frames (ORFs; Stothard, 2000) were predicted using the NCBI ORF finder program.^2^ Functional gene prediction was performed using domain-enhanced lookup time accelerated BLAST (Boratyn et al., 2012), and a cluster analysis was conducted through the cluster W method using Lasergene v14 (DNASTAR) with the Pfam database (Sonnhammer et al., 1997; El-Gebali et al., 2019). Genome assembly and annotation were conducted with the cooperation of Phyzen (Phyzen Genomics Institute, Seongnam, Korea), a bioinformatics company in Korea.

### 2.4. Phylogenetic analysis of common genes

The proteins in DfIV, including repeat elements (Reps), cysteine motif (Cys), vankyrins, polar residue-rich protein (PRRP), vinnexins, and neuromodulin N sequences, were used for alignment *via* Lasergene v14 (DNASTAR). Neuromodulin N was found to represent a distinct gene family in the DfIV genome. Unrooted phylogenetic tree construction with the protein sequences of Reps was performed by using MEGA 11.2 (Tamura et al., 2021) for comparison between the DfIV and DsIV genomes (Supplementary Figure 4A). The Jones–Taylor–Thornton (JTT) + Gamma-distributed (G) model was selected as the best-fit model and was used for maximum likelihood analysis with 1,000 bootstrap replicates. DfIV was distinguished from DsIV by the presence of colored (red color DfIV and green color DsIV proteins; cys-motifs, *n* = 10; vankyrins, *n* = 9; PRRP, *n* = 7; vinnexins, *n* = 6 proteins; *n* = copy number of each gene; Supplementary Figures 4B–E). Three duplicated copies of the vinnexins protein sequence were also used for the analysis, including vinnexin 3 (C12 and C13), vinnexin 4 (D6 and D11), and vinnexin 5 (12 and 13). In this study, 41 DfIV Rep protein sequences were identified, but one short protein sequence was excluded (*rep* 1–1, ULM71580.1) from the phylogenetic analysis.

### 2.5. Gene expression patterns in different hosts

Gene expression analyses in different hosts were performed to understand the molecular mechanisms of complex interaction (i.e., internal sequence homologies, number of segments, composition of sequences, integration motif and sites, and expression levels of genes). Gene expression patterns were analyzed in *D. fenestrale* and *P. xylostella* 1 and 5 days after inoculation. We used inoculated third and fifth instar *P. xylostella* on days 1 and 5, respectively, after inoculation. Five *P. xylostella* larvae were used as a biological replicate (pool of larvae). After 1 and 5 days of parasitization with *D. fenestrale*, total RNA was extracted from *P. xylostella* samples and the ovarian tissue of 30 parasitoids in each replicate (replicate consisting of ovaries from a pool of ovaries from several individuals) using an RNeasy Mini Kit (Qiagen, Hilden, Germany). The 2100 Bioanalyzer RNA 6000 NANO chip (Agilent) was used to confirm the quality of the RNA samples. RNAseq library preparation and main sequencing were performed following the procedures described in section 2.3. RNA-seq data were trimmed using Trimmomatic v0.36 (Bolger et al., 2014). Parameters of *p*-value < 0.05 and|log2 (fold change)| > 1 were used to determine significantly differentially expressed transcripts (DNASTAR; EdgeR). Statistical analysis was performed based on read counts or fragments per kilobase million values. Trimmed and assembled RNA-seq data were mapped (BWA tools, version 0.7.10) to DfIV genome (Supplementary Table 1), and the *P. xylostella* genome (GCA_000330985.1) was also used for comparative analysis.

### 2.6. Genome-wide survey of DfIV host genome integration motifs

After 1 day of parasitization with *D. fenestrale*, gDNAs were extracted from *P. xylostella* samples hosting parasitoid eggs. A single larva was used as a biological replicate, and three larvae genomes were re-sequenced. Library preparation and genome sequencing were performed following the procedures for virus genome sequencing described in section 2.3. Resequencing data were trimmed to remove the adapter and low-quality reads using Trimmomatic v0.36 (Bolger et al., 2014) and the trimmed high-quality data were mapped to the genome using the Burrows–Wheeler Aligner (BWA) software (version 0.7.17, Li and Durbin, 2009) with default parameters. SAMtools software (version 1.15, Li et al., 2009) was used to convert mapping results into the BAM format and to remove the unmapped and non-unique reads. The Picard package (version 2.27)^3^ was used to filter duplicated reads. Trimmed re-sequence data were mapped to the DfIV reference genome, and the *P. xylostella* genome (GCA_000330985.1) was used for comparison. The investigated integration motifs in the host genome revealed microhomologies (≥15 read count/number of repeat sequences, and 10-, 20-, and 50-motif length) between the host genome and the DfIV circles at integration breakpoints. If an integration motif matched a *P. xylostella* genome read count of 15 bp, it was not counted. Microhomologies (threshold level ≥ 15 read count/ number of repeat sequences, and 10-, 20-, and 50-motif length, see Supplementary Table 4) were revealed between the host genome and the DfIV circles at integration breakpoints.

### 2.7. Data availability

The DfIV genome data (62 segments) were deposited in the NCBI database under accession numbers MZ129220–MZ129281. The DsIV genome sequences (47 segments) were previously deposited by Wang et al. (2021) under the GenBank accession numbers KF156214–KF156260, and the new segment identified in this study (segment no. 48) was deposited under GenBank accession number MZ129282. Three segments of Saesbyeol virus genome data were deposited: large (MH698456), medium (MH823676), and small (MH698457).

## 3. Results

### 3.1. Virion morphology of DfIV with transmission electron microscopy

**Figure 1:**
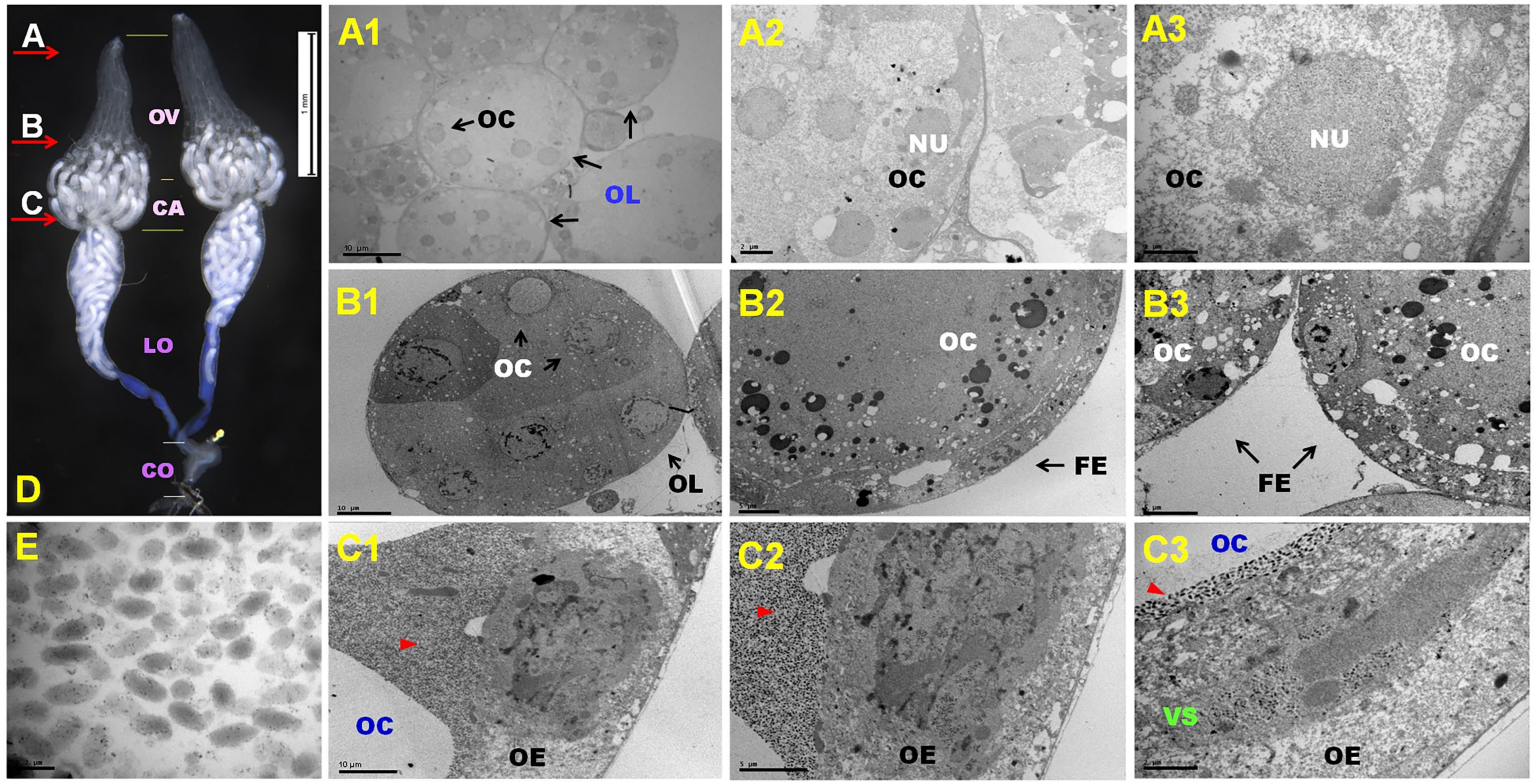
**(A–E)** Representative ovary structure of *Diadegma fenestrale* (D); red arrow indicates the dissection position and direction of the cutting sites **(A–C)**. The reproductive organ comprised four major parts: ovary (OV), calyx (CA), lateral oviduct (LO), and common oviduct (CO). Cutting site **(A)**: oogenesis at the germarium of the *D. fenestrale* ovary **(A1–A3)**. A single ovary contained about 10 ovarioles. More than five immature oocytes (OC) were located in each ovariole (OL) **(A1)**. A detailed view of the ovariole is shown in **(A2)**. Five oocytes and their nuclei (NU) were observed in an ovariole. A detailed view of the oocyte is shown in **(A3)**. Cutting site **(B)**: OCs in vitellogenesis are surrounded by the follicular epithelium (FE). Six OCs were present in an OL **(B1)**. A detailed view of the OCs is shown in **(B2,B3)**. Cutting site **(C)**: OCs surrounded by virion particles at the calyx area after vitellogenesis. The ovarian epithelium (OE) contains virogenic stroma (*VS*) and releases the virion particle to the calyx chamber. Red triangles indicate the presence of DfIV particles **(C1,C2)**. DfIVs were located within the OE with OCs **(C3)**. Newly growing oocytes were abundant at the ovarian calyx and lateral oviducts (**D**). DfIV showed a double membrane envelope, but the inner membrane could not be distinguished clearly **(E)**.

DfIV morphology was observed within the nuclei of calyx epithelial cells in female reproductive organs (Figure 1). To confirm the presence of DfIV in the ovary, ultrathin cross-sections of the distal ovary, proximal ovary, and calyx regions were prepared and examined using transmission electron microscopy (TEM; Figures 1A–C, and E). Each ovary comprised more than 10 ovarioles, each containing developing oocytes (Figure 1A1). Nuclei were observed inside the oocytes (Figures 1B1,C1). More than five developed oocytes were observed (Figure 1A2). Each oocyte had a relatively large nucleus surrounded by follicular epithelium (Figures 1B2,C2). DfIV particles were identified within the calyx, alongside immature oocytes (Figures 1A3,B3). Each ovarian epithelial cell contained a large nucleus with highly stained materials. The viral particles were ellipsoidal, with an average size of 246.45 nm × 1.20 nm (long axis) and 108.95 nm × 1.64 nm (short axis), and possessed double membranes in the form of bilayer envelopes that assembled to form an intranuclear virogenic stroma (Figure 1C3). Each pair of ovaries was filled with fully grown oocytes at the ovarian calyx; lateral oviducts are shown in Figure 1D. Blue-colored materials were also detected in the lumen, representing fully grown oocytes. Stem cells and early immature oocytes were observed in the distal ovariole region.

### 3.2. Genomic organization and annotation of DfIV

The analyzed genome size determined by gel electrophoresis may be skewed due to the poor quality of separation of supercoiled genome segments and differences in the abundance of genome segments. Therefore, electrophoresis confirmed only that the DfIV genome comprised segments of various sizes, but the exact number and size of segments could not be determined (Supplementary Figure 1). The Illumina-based genome sequence confirmed the precise number (*n* = 62, number of segments) and size of segments (1.4–6.6 kb; Supplementary Table 2).

A total of 20,810,524 bp was sequenced *via* Roche 454 from 51,684 reads and assembled to approximately 120 contigs. Following the Illumina-based genome sequence, 62 circular DNA segments were assembled with an approximate total size of 247 kb (Supplementary Table 2). A total of 126 ORFs were predicted, but three ORF gene sequences were located in different loci of gene segments and had identical or slightly modified sequences from the original. Therefore, we trimmed the overlapping sequences and predicted a total of 123 ORFs (Table 1). The complete genome size, GC content, number of segments, and number of genes were compared between DfIV and other PDVs. Genome size and GC content were highly similar among DfIV and ichnoviruses of other Campopleginae family parasitoid hosts, *H. fugitivus* ichnovirus (HfIV) and *C. sonorensis* ichnovirus (CsIV). *Glypta fumiferanae* ichnovirus (GfIV) and *Apophua simplicipes* ichnovirus (AsIV; Banchinae) showed slight differences in genome size and number of segments when compared with DfIV. The total genome size of AsIV was estimated to be 300 kb, similar to the 292 kb reported for GfIV (Lapointe et al., 2007) and with comparable gene families and variation in a number of genome segments (AsIV > 132 and GfIV 105). However, the numbers of segments (15–132) and genes (27–186) were highly variable (Table 1). The DfIV genome segment size ranged from 1.5 to 8.2 kb with a median of 3.77 kb. DfIV genome characteristics were different from those of bracoviruses, including CcBV, MdBV, and CvBV (Table 1).

**Table 1:** List of comparable data from diverse ichnoviruses and bracoviruses regarding genome sequence length, gene segment abundance, and number of genes.

Organism	Size (Kb)	GC%	No. of segments (size ranges, Kb)	No. of genes	Reference/GenBank ID
**Ichnovirus (Campoplegine)**					
*Diadegma fenestrale* ichnovirus, DfIV	247	43	62 (1.5–8.2)	123	This study
*Diadegma semiclausum* ichnovirus, DsIV	208	43	48 (2.8–7.7)	107	Wang et al. (2021) and this study
*Hyposoter fugitivus* ichnovirus, HfIV	246	43	56 (2.6–8.9)	135	Tanaka et al. (2007)
*Campoletis sonorensis* ichnovirus, CsIV	247	41	22 (6.1–19.6)	106	Webb et al. (2006)
*Hyposoter didymator* ichnovirus, HdIV	263	43	50 (2.5–3.6)	134	Doremus et al. (2014)
*Tranosema rostrale* ichnovirus, TrIV	250	42	20 (4.1–10.1)	>89	Tanaka et al. (2007)
**Ichnovirus (Banchine)**					
*Glypta fumiferanae* ichnovirus, GfIV	292	37	105 (<1.0–5.2)	103	Djoumad et al. (2013)
*Apophua simplicipes* ichnovirus, AsIV	300	~	>132 (~1.0–4.0)	186	Djoumad et al. (2013)
**Bracovirus**					
*Cotesia congregata* bracovirus, CcBV	568	34	30 (5.0–40.0)	182	Chevignon et al. (2014)
*Microplitis demolitor* bracovirus, MdBV	185	34	15 (3.6–34.3)	60	Beck et al. (2007)
*Cotesia vestalis* bracovirus, CvBV	540	35	35 (2.6–39.2)	157	Chen et al. (2011)
*Glyptapanteles indiensis* bracovirus, GiBV	508	36	29 (9.7–39.0)	27	Desjardins et al. (2007)
*Glyptapanteles flavicoxis* bracovirus, GfBV	594	36	29 (3.8–50.7)	27	Desjardins et al. (2008)
*Cotesia congregata* bracovirus, CcBV	568	34	36 (4.9–41.6)	174	GCF_000844405.1
*Diolcogaster facetosa* bracovirus, DfIV	787	35	31 (3.8–50.7)	112	ASM283394v1
*Cotesia plutellae* bracovirus, CpBV	351	35	24 (6.4–23.7)	124	Choi et al. (2009)

### 3.3. Comparative features of DfIV and DsIV ORFs

**Figure 2:**
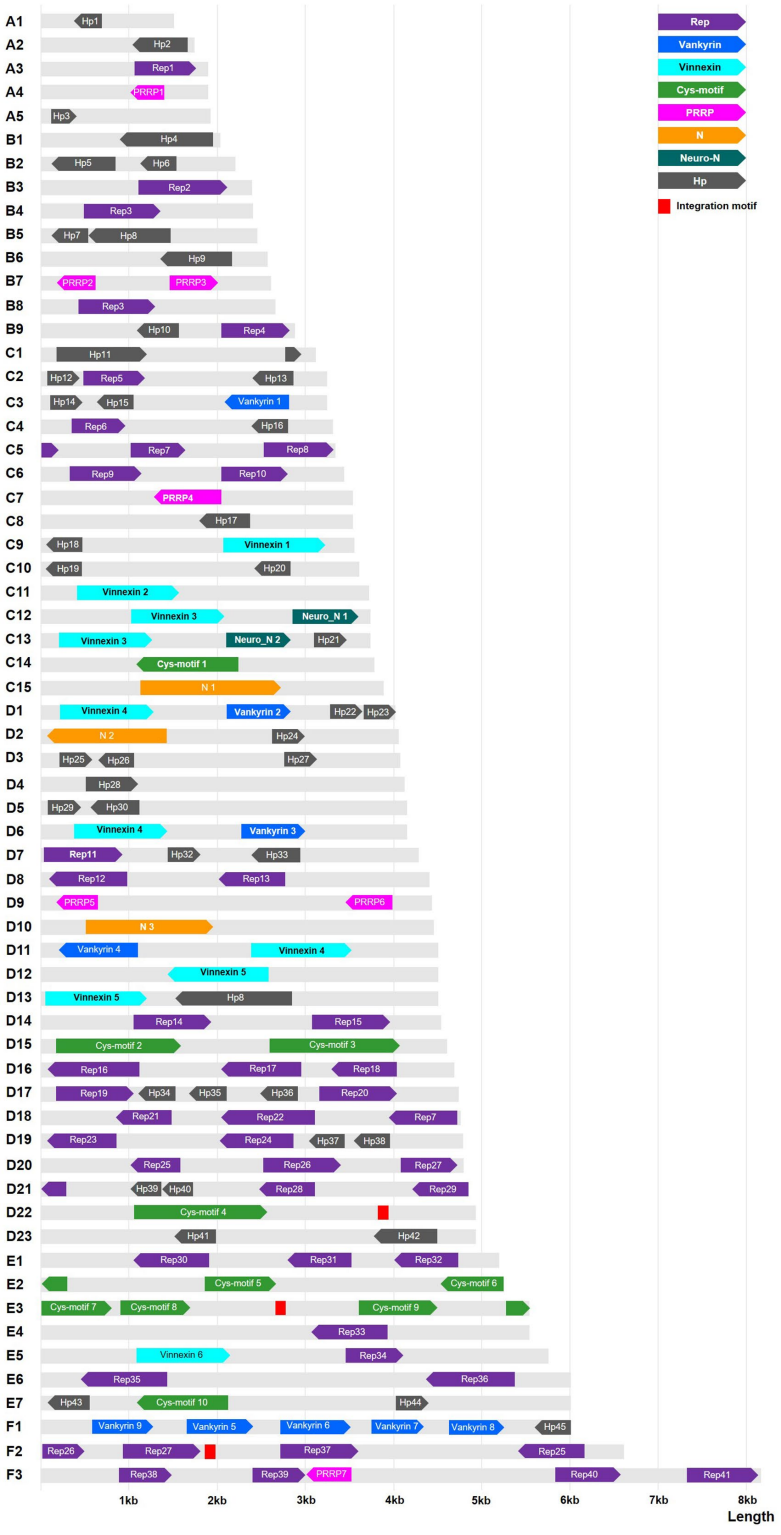
Graphical representation of the DfIV genome and its annotated genes, with 62 non-redundant (NR) circular genome segments shown as linear molecules. DfIV genome segments were 1.5–8.2 kp in length. Colored boxes show the sizes (length) and arbitrary locations of gene families, with directions indicated by the arrowheads on each box. Red boxes indicate integration motifs of the lepidopteran host genome. Gray regions represent non-coding DNA. Hp, hypothetical protein; PRRP, polar residue-rich protein 1; Rep, repeat element protein; Neuro-1, neuromodulin N1.

In the DfIV genome, we identified 123 ORFs/genes, including *rep* (*n* = 41), *cys* (*n* = 10), *vankyrin* (*n* = 9), *vinnexin* (*n* = 6), *PRRP* (*n* = 7), *neuromodulin* N1 (*n* = 2), and unassigned ORFs (*n* = 45), which we numbered based on the genome segments of DfIV (*N* = 62) and their segment-specific order. Forty-one of these putative ORFs overlapped in at least one direction (Figure 2). Approximately one-third (21/62; A1–5; B1, 3, 4, 6, 8; C1, 7, 8, 11, 14, 15; D4, 10, 12, 22; and E4) of the DfIV genome segments contained only one putative ORF (Figure 2). In the DsIV genome, 103 ORFs/genes were annotated, including *rep* (*n* = 36), *cys* (*n* = 8), *vankyrin* (*n* = 6), *vinnexin* (*n* = 7), *PRRP* (*n* = 7), *N-protein* (*n* = 3), and unassigned ORFs (*n* = 36). The genome of DsIV had already been sequenced (*N =* 47 segments; Wang et al., 2021), but our analysis identified an extra segment (segment number 48, GenBank accession no: MZ129282.1) containing three hypothetical proteins (ULM71713, 171; ULM71712, 109; and ULM71711, 79 amino acids). No *neuromodulin* N genes were identified in DsIV. We aligned the two gene sequences of *neuromodulin* N from DfIV with those of other closely related three sequences (DfIV_Neuromodulin_N1_C12, DfIV_Neuromodulin_N2_C13, HdIV_U2 [AIK25642], HfIV_b7.1 [YP001031227], and DsIV_vinnexin1 [AHY21952]), based on BLAST sequence similarity searches and observed indels on the aligned gene sequences (Supplementary Figure 2).

**Figure 3:**
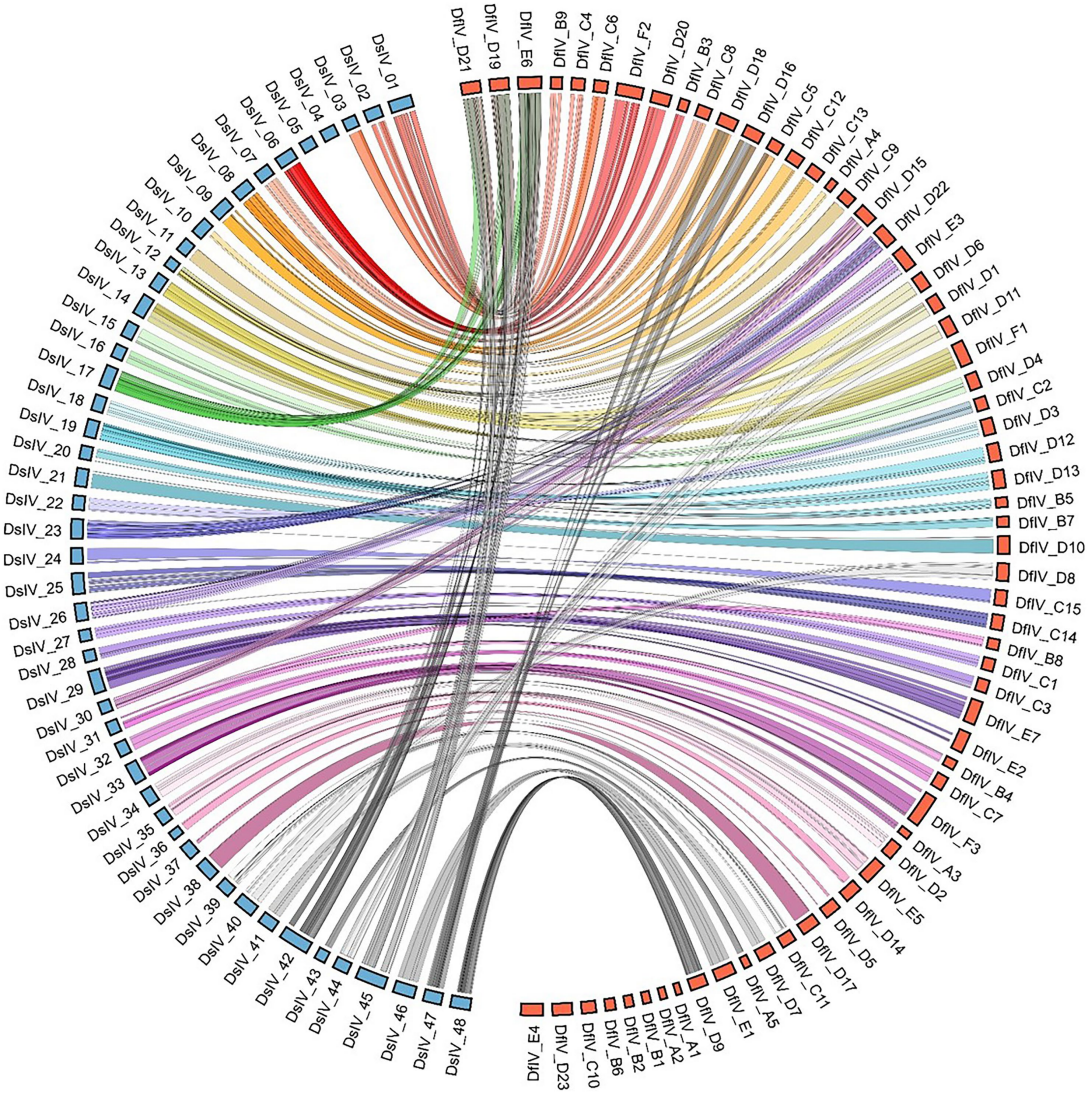
Comparison of sequence similarity between 62 genomic segments of DfIV (red) and 48 genomic segments of DsIV (blue) based on BLAST results. Eight specific segments of DfIV and two of DsIV were identified. The DsIV genome was re-sequenced using the AVRDC strain as a template (Taiwan strain, Kim et al., 2015). Comparative analyses revealed a high degree of synteny between loci coding for DfIV and DsIV proviral segments, as well as flanking DNA.

Whole-genome sequence similarity was analyzed and visualized of corresponding genomic areas between 62 genomic segments of DfIV and 48 genomic segments of DsIV (Figure 3). The sequence similarity between DfIV and DsIV was high, ranging from 85% to 96% with an average of 90% similarity. Nine DfIV-specific segments (A1, A2, B1, B2, B6, C10, D23, E2, and E4) and two DsIV-specific segments (4 and 5) were non-homologous. Phylogenetic analysis of commonly observed proteins (Rep, Cys, PRRP, Vankyrin, and Vinnexin) in DfIV and DsIV confirmed a high degree of similarity (Supplementary Figure 3). The diversity of *rep* genes (41 members) was higher than that of *cys, vankyrin, PRRP,* and *vinnexin* genes (10, 9, 7, and 6 members, respectively). Therefore, we developed an unrooted phylogenetic tree of *rep* genes (Supplementary Figure 4A). Maximum likelihood analysis was conducted based on the deduced amino acid sequences of DfIV and DsIV *rep* proteins (>150 aa). In addition, *reps* were found in all ichnoviruses. The 40 protein sequences used for phylogenetic analyses were well cladded and clustered. Four commonly found ichnovirus genes, including *cys*, *PRRP*, *vankyrin*, and *vinnexin,* were downloaded from NCBI to construct phylogenetic trees (Supplementary Figures 4B–E) and clarify the evolutionary origins of ichnovirus and bracovirus lineages. The most abundant (41 members) *rep* gene family was encoded in 20 different segments. However, three members (7, 25, and 27) existed as duplications in different segments, including *rep* 7 in C5 and D18, *rep* 25 in F2 and D20, and *rep* 27 in F2 and D20 segments. Furthermore, one segment (A3) contained two rep genes (*rep*1-1 and *rep*1-2; see accession no MZ129222.1, segment A3). Two copies of DfIV *vinnexins* were observed in the DfIV genome, *vinnexin* 3 (segments C12 and C13), *vinnexin* 4 (segments, D1 and D6), and *vinnexin* 5 (segments 12 and 13; Figure 3). No duplicated genes were observed in DsIV.

### 3.4. Gene expression patterns in different hosts

**Figure 4:**
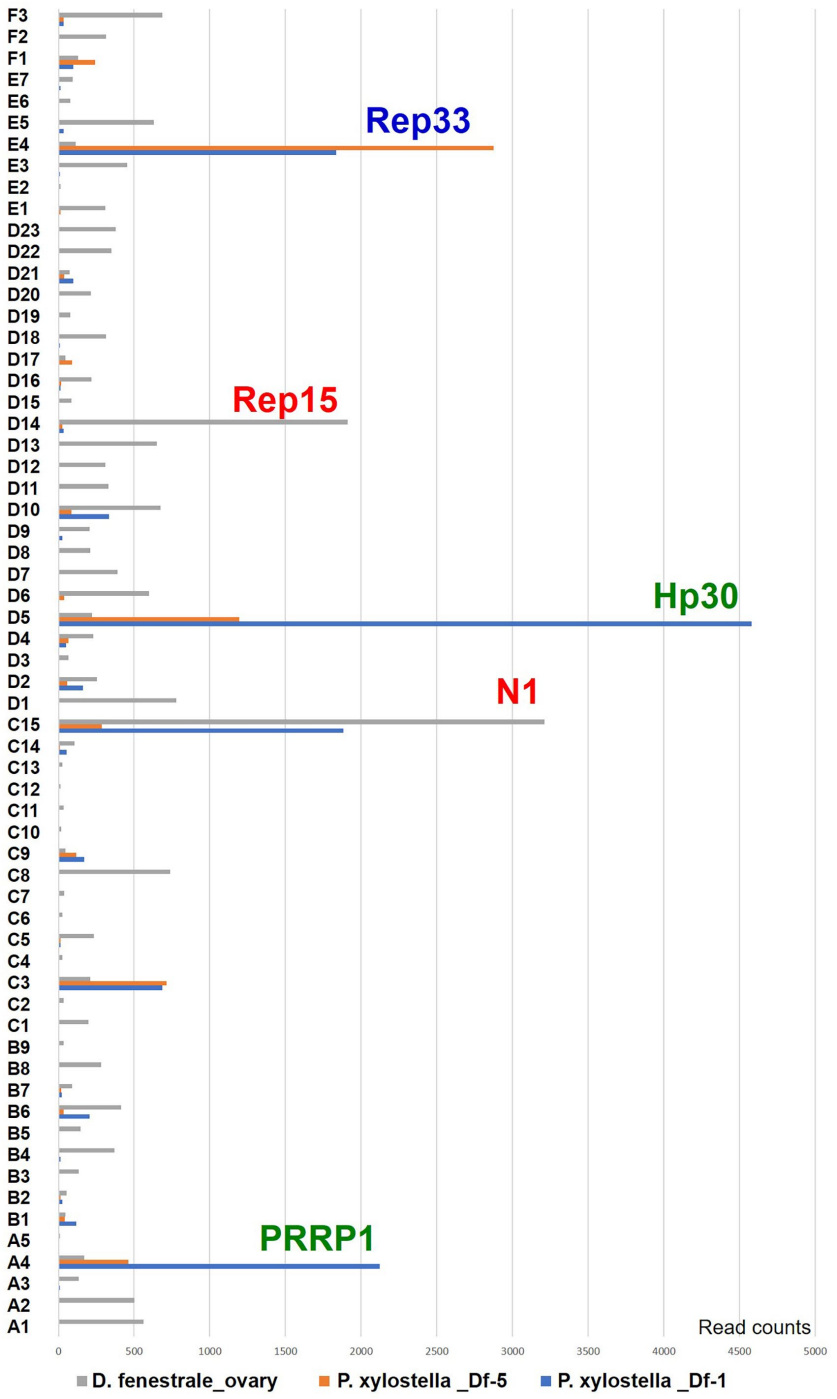
Gene expression pattern in two different hosts (hymenopteran host, *Diadegma fenestrale,* and lepidopteran host, *Plutella xylostella*) and developmental stages of *P. xylostella* based on RNAseq. The segments are expressed in the ovaries of hymenopteran host, *D. fenestrale*; only two segments (C15 and D14) were highly expressed in *D. fenestrale* ovaries. Five segments (E4, D5, C15, C3, and A4) were differentially expressed according to the developmental stage of *P. xylostella* (1 and 5 days after parasitization by *D. fenestrale*; third and fourth larval instar).

Gene expression patterns were examined in different viral hosts and life stages (hymenopteran host, *D. fenestrale;* lepidopteran host, *P. xylostella*). A total of 62 genome segments were expressed in *D. fenestrale* ovaries, and segments C15 and D14 were particularly highly expressed (Figure 4). Five segments (E4, D5, C15, C3, and A4) were differentially expressed in *P. xylostella* of different developmental stages (third and fifth instar). The most highly expressed gene in the third instar *P. xylostella* was hypothetical gene Hp 30 in segment D5 (*P. xylostella* _Df-1, 1 day after parasitization by *D. fenestrale*) and that in fifth instar larvae was Rep 33 in segment E4 (*P. xylostella Df*-5, 5 days after parasitization by *D. fenestrale*). The highest expressed gene in *D. fenestrale* was N1 in segment C15, followed by Rep15 in segment D14.

### 3.5. Genome-wide survey of DfIV host genome integration motifs

A *P. xylostella* genome was re-sequenced 1 day after parasitization by *D. fenestrale,* and the resequencing results of the host genome integration motif are shown in Supplementary Table 3. PDV (DfIV) cannot replicate within its lepidopteran host, *P. xylostella*, but genes are integrated and expressed throughout the parasitism. Genome integration is also dependent on host immunity and physiology under high selection pressure because DNA integration requires the parasitoid’s success. Only virus encapsidation and expression sequences in the genome of the lepidopteran host (*P. xylostella*) are required to be present on the DNA circles (Drezen et al., 2003). The homologous recombination was evidenced between left and right direct repeat junctions (DRJs) to combine one DRJ sequence. The presence of multiple repeats that differ in sequence and position suggests that homologous recombination may result in a mix of overlapping and/or nested segments (Legeai et al., 2020). However, IV replication and integration mechanisms in the host genome are largely unknown. In this study, host genome integration motifs were compared between the DfIV and *P. xylostella* genomes, and two complementary conserved motifs were surveyed at their breaking sites. The general mechanism of motif integration was as follows: (1) The homologous region between host and viral genomes ranged from 36 to 46 bp, and the integration motifs existed on both sides of DfIV and *P. xylostella*; (2) each homologous site on the host and viral genome was integrated into the host genome; (3) using the process of homologous recombination (Legeai et al., 2020), the homologous region and DfIV genome with integrated motifs were combined into the host genome (Supplementary Figure 5). Across three biological replicates of *P. xylostella* genome (DBM-Df-1, DBM-Df-3, and DBM-Df-7), resequencing results are shown in Supplementary Table 3, and only three genomic segments (DfIV-D22, -E3 and -F2) were predicted to be integrated. Two genomic segments (D22 and E3) were integrated into one *P. xylostella* chromosome, but F2 integration motifs were found at five *P. xylostella* chromosome sites (F2–1 to F2–5; Supplementary Table 4). Of the investigated integration motifs in the host genome, the D20 segment was not matched to the *P. xylostella* genome within the threshold parameters, so it was not counted (data not shown). DfIV genomic segment replication was not detected in the lepidopteran host (data not shown).

### 3.6. New virus identification from *Diadegma fenestrale* and *Plutella xylostella*

In this study, one new virus was found in *D. fenestrale* and *Plutella xylostella* through transcriptome analysis; this was denoted as “Saesbyeol virus.” The aggregate genome sequence length of Saesbyeol virus was 14, 447 bp, and the genome contained three independent segments, large, L (MH698456, 6,987 bp), small, S (MH698457, 1,912 bp), and medium, M (MH823676, 5,548 bp), similar to Bunyaviridae family, which is divided into three segments called L, M, and S based on their segment size (Amroun et al., 2017). The sequence MH698456 contains a conserved protein domain, based on NCBI’s conserved domain database (CDD) search, and it encodes a bunyavirus RdRP (RNA-dependent RNA polymerase). MH823676 BLAST matches are viral sequences only, of which most are glycoproteins of bunyaviruses. In addition, MH698457 matches virus sequences only, all bunyavirus nucleocapsid proteins. It seems very likely that the three segments are derived from the same virus as they match sequences of bunyaviruses which typically have a genome consisting of several segments. The genome assembly (ASM412811v1) was deposited to the NCBI database.^4^ The function of Saesbyeol virus and its interactions with DfIV and its hosts (*D. fenestrale* and *P. xylostella*) should be investigated in future studies.

## 4. Discussion

PDVs are obligatory symbionts of thousands of endoparasitoid wasp species and are essential for their successful parasitism by modulating host immunity (Ignesti et al., 2018). Here, TEM analysis showed that the viral replication was likely to occur in the ovarian calyx, with replicated viral particles released into the oviduct lumen. In PDV-harboring wasps, the ovary is well developed in the calyx region where the PDVs are replicated (Volkoff et al., 1995). DfIV morphogenesis was observed within the nuclei of calyx epithelial cells in female reproductive organs (Figure 1), which is likely to be present in other closely related PDV (Lorenzi et al., 2022). Furthermore, ichnoviruses and bracoviruses share a common life cycle but are morphologically different. Unique morphological characteristics include a layer of cell membrane that discriminates ichnoviruses from bracoviruses (Lapointe et al., 2007). Bracoviruses have cylindrical nucleocapsids surrounded by a single envelope, while ichnoviruses have lenticular nucleocapsids with two membranous envelopes (Krell, 1987). The morphology was detected in this study, though the inner membrane of DfIV was not distinguished.

The DfIV genome sequence was too complicated to be fully sequenced because of segmentation and internal sequence homologies between segments (Webb, 1998; Federici and Bigot, 2003). Unequal replication of different segments is a characteristic of PDVs (Beck et al., 2007), though little is understood about the exact mechanism explaining this unique kind of viral replication. The resulting 65 genomic segments were re-sequenced using the Illumina platform with a high depth of genome sequence coverage, narrowing the results to 62 genomic segments. However, in ichnoviruses, an unequal number of segments can be partially explained by a segment nesting process (Cui and Webb, 1997; Kadash, 2000). Ichnovirus genome segments have been observed to range in size from 2 to 28 kb (Webb and Cui, 1998), with estimated genome sizes from 75 to 250 kb. In accordance with this, the DfIV genome segments analyzed here ranged from 1.5 to 8.2 kb with a total genome size of approximately 247 kb.

For most parasitoids, successful parasitism depends on ecological, biological, and anthropological factors, and molecular mechanisms are not well understood (Vinson and Iwantsch, 1980). However, it has been suggested that some parasitoids can extend their host survival as a consequence of coevolution between parasitoids and their hosts. For ichnovirus-harboring parasitoids, successful parasitism is dependent on the expression of certain ichnovirus genes exceeding a threshold value (Doremus et al., 2014). In one study, the differential expression of two cysteine proteins, CsIV VHv1.1 and VHv1.4, influenced lepidopteran hosts to ensure successful parasitism by *C. sonorensis* (Cui et al., 2000; Kim, 2005). Therefore, we focused on the expression patterns of common (cysteine motif protein, *cys;* viral ankyrin, *vankyrin*; viral innexin, *vinnexin*; and unassigned genes) and novel (*PRRP*, *N* gene, and *neuromodulin N*) PDV gene families that may contribute to host immune response. The diversity of gene expression was dependent on the nature of the host species (permissive hosts, where the host cellular immune response cannot eliminate parasitoid eggs, and non-permissive hosts, where the host cellular immune response can eliminate parasitoid eggs).

In our previous study (Kim et al., 2015), *D. fenestrale* was observed to prefer *P. operculella* to *P. xylostella* hosts, according to ovipositional and survival rate analyses. DfIV gene expression in *P. operculella* was higher than that in *P. xylostella* after parasitization by *D. fenestrale*, suggesting that higher DfIV gene expression promoted the survival of parasitoids. However, the exact mechanisms of host preferences have not yet been explored. Moreover, qRT-PCR analysis results of gene expression were observed after parasitization by *D. fenestrale*, but many DfIV genes were not expressed, including *PRRP*, neuromodulin N1, hypothetical genes (Hp), and several *rep* genes (identified *rep* genes in the present research, *n =* 41; *rep* genes identified in previous research, *n =* 28; Kim et al., 2015). Interestingly, Rep33 gene was more highly expressed in segment E4 of DfIV at 5 day post-parasitization than at 1 day post-parasitization of *P. xylostella* (Figure 4). This result indicated that Illumina-based analysis yielded better resolution than the Roche 454 with the qRT-PCR strategy used previously (Kim et al., 2015). In an earlier study, the segments of PDV that encode virulence genes were demonstrated to be transcribed in the parasitized hosts but not in the wasps themselves (Strand and Burke, 2013). This raises a question as to why a large amount of N gene transcript, which is known to be involved in host immune suppression, was expressed in the ovary of the parasitoid wasp. One possible hypothesis is that expression of a single gene may vary depending on the interaction with the host, but further research is still needed.

Five gene families (*rep, cys, PRRP, vankyrins,* and *vinnexins*) were identified in both DsIV and DfIV genomes, as previously reported in many studies (Webb et al., 2006; Tanaka et al., 2007; Doremus et al., 2014; Wang et al., 2021). Among these families, *rep* was the most abundant in DfIV, with 41 predicted members. *Rep*s have been previously observed in several ichnoviruses, including HfIV, CsIV, and TrIV (Galibert et al., 2006; Rasoolizadeh et al., 2009). Although the function of *rep* genes has not yet been fully elucidated, their conservation and abundance in viral genomes indicate that they play an important role in viral maintenance (Galibert et al., 2006). The second most common family, *cys-motif,* was represented by 10 members in DfIV. The *cys-motif* family is found in other ichnoviruses (eight members in DsIV) but not in bracoviruses (Zhang and Turnbull, 2021). Two *cys-motif* genes in CsIV (VHv1.1 and VHv1.4) affect the hemocytes so that the cellular immune system would be suppressed, to the benefit of the hymenopteran insect (Kim, 2005; Ye et al., 2018). Therefore, *cys-motif* genes in DfIV may have similar molecular functions (Kim, 2005). Genes of the third most represented family, *PRRP*, contain approximately 63–77% polar amino acids (Tanaka et al., 2005), but their function is unknown (Tanaka et al., 2007). The fourth most abundant family, *vankyrins*, included nine members in DfIV and six in DsIV. Unlike other ichnovirus genes, *vankyrins* are also encoded in bracoviruses (Webb, 1998). The nuclear factor kappa-light-chain-enhancer of activated B cells (NF-κB) is an important transcription factor that is expressed in almost all cells. The CsIV vankyrin proteins are thought to be involved in the suppression of NF-κB activity during immune responses and/or parasitized host development. The fifth gene family, *vinnexins*, exhibits homology with the insect gap junction innexin genes and was predicted in DfIV with six members and in DsIV with seven. Vinnexin is a highly conserved protein in ichnoviruses and may interfere with host cell–cell communication (Phelan et al., 1998), alteration of cytoskeletal networks (Hasegawa et al., 2020), host cell encapsulation, molting, and larval maturation (Zhang and Turnbull, 2021). Here, the phylogenetic relationships of these five DfIV and DsIV gene families (*rep, cys, PRRP, vankyrin*, and *vinnexin*) were compared and found to be closely related. Phylogenetic analyses can reveal important information related to the evolutionary origin of viruses (Federici and Bigot, 2003). In addition, other canonical ichnovirus genes, such as *neuromodulin N*, and 45 unassigned genes were predicted in DfIV. Neuromodulin is also referred to as growth-associated protein 43 (Gap-43) and is considered a “growth” or “plasticity” protein in humans because it is expressed at high levels in neuronal growth cones during development (Apel et al., 1990; Benskin et al., 2009) and interacts with many proteins. The neuromodulin gene is known as a gap junction gene and may be related to immune suppression (Glatz et al., 2004). Until now, no functional information has been elucidated regarding viral neuromodulin, but we observed moderate sequence similarity with both viral *innexin* and *vinnexin*. Moreover, neuromodulin genes identified in this study showed similarity to HdIV_U2 and HfIV_b7.1, genes from generalist ichnoviruses in *Hyposoter didymator* and *H. fugitivus* (Doremus et al., 2014). Some indels in protein-coding regions (i.e., neuromodulin genes, unknown function based on BLAST) of DfIV may be beneficial for understanding evolutionary processes (Sung et al., 2016).

Host integration motifs of DsIV circles have been observed in the parasitized *P. xylostella* genome (Wang et al., 2021), and similar integration motifs of DfIV were observed here. The precise mechanism of genome integration remains unknown, and the exact functions of the integration motif, integration sites, and homologous sequences in the DfIV genome are still poorly understood. Previous research demonstrated that PDVs can exist in host cell episomes with full capability to express virulence genes or immunosuppressive agents (Beck et al., 2011; Burke and Strand, 2012), promoting virus survival. The integration process into the host genome appears to be site-specific and is mediated by the wasp genome; it is also dependent on the integration machinery of specific hosts (Burke and Strand, 2012). The ability of segments to integrate into host cells explains how PDV gene products are expressed in hosts for long periods (Beck et al., 2011; Burke and Strand, 2012). In a past study, Wang et al. (2021) showed that DsIV genome segments or circles existed in *P. xylostella* host hemocytes and were integrated into the host genome either conservatively or randomly. However, they were unable to determine the precise location of the repeat motif and homology sequences, nor could they identify the position of the virus and host genomes. Here, we compared host integration motifs and homology sequences between DfIV and *P. xylostella* genomes and proposed a mechanism for their integration based on previous reports on HdIV and CsIV (Legeai et al., 2020). Further research should be conducted to enhance the understanding of integration motifs and their patterns in DfIV and its host. According to our present findings, some DfIV genome segments can integrate into the *P. xylostella* genome, as suggested in previous research (Kim et al., 1996; Volkoff et al., 2002). Because many lepidopteran hosts can be parasitized by generalists, DfIV is thought to have a high probability of integration in various species (Doucet et al., 2007) while accelerating the evolution of the viral genome (Kim et al., 1996; Volkoff et al., 2002). Furthermore, we observed microhomologies (≥15 read count, see Supplementary Table 4) between the *P. xylostella* host genome and the DfIV circles at integration breakpoints, suggesting a potential mechanism similar to that of human papillomavirus integration (Hu et al., 2015). Further research is needed to determine how host genome segments can become complete circles.

This study did have some limitations. Both DfIV and DsIV have been maintained under lab conditions for over a decade with their hymenopteran hosts. It is, therefore, possible that these PDVs have begun to develop unique evolutionary lineages. Our findings would be reinforced by employing purer or more recent lines of DfIV and DsIV. Alternatively, we can compare our findings against results from Chinese and Taiwanese DsIV strains to understand strain-specific variations. Further investigations should focus on long-term evolutionary consequences of coevolution (natural selection-driven reciprocal evolutionary changes) between a virus and its hosts, analysis of known functional genes, putative functional analysis of unknown genes, and gene expression analysis in viral hosts. Our present findings provide insight for further investigations regarding viral evolution and virus–host coevolution.

## Data availability statement

The datasets presented in this study can be found in online repositories. The names of the repository/repositories and accession number(s) can be found in the article/Supplementary material.

## Author contributions

JK and YK: conceptualization and experimental design. M-MR and JK: software and writing the original draft. JK, M-MR, A-YK, SR, MK, and YK: writing, reviewing, and editing. JK, A-YK, SR, MK, and YK: formal analysis and validation. JK, SR, MK, and YK: resources and editing. JK: supervision and project administration. All authors contributed to the article and approved the submitted version.

## Funding

This study was supported by the Basic Science Research Program through the National Research Foundation of Korea (NRF), funded by the Ministry of Education (NRF-2021R1A6A1A03044242), Republic of Korea.

## Conflict of interest

The authors declare that the research was conducted in the absence of any commercial or financial relationships that could be construed as a potential conflict of interest.

## Publisher’s note

All claims expressed in this article are solely those of the authors and do not necessarily represent those of their affiliated organizations, or those of the publisher, the editors and the reviewers. Any product that may be evaluated in this article, or claim that may be made by its manufacturer, is not guaranteed or endorsed by the publisher.

## Footnotes

^1^ https://www.ncbi.nlm.nih.gov/nuccore/?term=txid993492[Organism:noexp]

^2^ https://www.ncbi.nlm.nih.gov/orffinder/

^3^ http://broadinstitute.github.io/picard/

^4^www.ncbi.nlm.nih.gov/search/all/?term=Saesbyeol%20virus%20, accessed date 02 August 2022.
