# Supplementary material for: Genome, host genome integration, and gene expression in Diadegma fenestrale ichnovirus from the perspective of coevolutionary hosts
Source: Front Microbiol. 2023 Feb 17;14:1035669. doi: 10.3389/fmicb.2023.1035669 (PMC9981800; doi:10.3389/fmicb.2023.1035669)
Supplement: Supplementary file 1 [file Table_6.DOCX]

Supplementary Material

Genome, Host Genome Integration, and Gene Expression in Diadegma fenestrale Ichnovirus from the Perspective of Coevolutionary Hosts

# Juil Kim ^1, 2*,^ Md-Mafizur Rahman^3^, A-Young Kim^4^, Ramasamy Srinivasan^5^, Min Kwon^6^, Yonggyun Kim

*** Correspondence:** Corresponding Author: forweek@kangwon.ac.kr

# Supplementary Figures and Tables

**Supplementary Table 1**. RNAseq data sets with comparison of raw and trimmed values

| **RNAseq raw data** | | | | | | |
| --- | --- | --- | --- | --- | --- | --- |
| Parasitization Category | Sample | Total Bases | Read Count | GC (%) | ^a^Q20 (%) | ^b^Q30 (%) |
| ^#^Df ovary | Df_ovary-1 | 2,891,685,550 | 28,630,550 | 52.67 | 97.75 | 96.04 |
|  | Df_ovary-2 | 2,674,833,096 | 26,483,496 | 53.28 | 97.31 | 95.26 |
|  | Df_ovary-3 | 3,002,364,784 | 29,726,384 | 52.62 | 97.31 | 95.26 |
| *DBM-1 (without P) | DBM-1-2 | 2,847,914,372 | 28,197,172 | 51.07 | 98.75 | 96.32 |
|  | DBM-1-3 | 2,189,786,252 | 21,681,052 | 51.66 | 98.74 | 96.30 |
|  | DBM-1-4 | 2,207,621,438 | 21,857,638 | 51.38 | 98.68 | 96.16 |
| DBM-5 (without P) | DBM-5-1 | 2,029,617,018 | 20,095,218 | 53.48 | 98.85 | 96.55 |
|  | DBM-5-2 | 2,411,787,888 | 23,879,088 | 54.87 | 98.80 | 96.40 |
|  | DBM-5-3 | 1,883,908,560 | 18,652,560 | 52.63 | 98.77 | 96.40 |
| DBM-1 (one day after P) | DBM-Df-1-1 | 2,436,502,992 | 24,123,792 | 50.82 | 98.62 | 96.01 |
|  | DBM-Df-1-3 | 2,183,950,472 | 21,623,272 | 52.12 | 98.71 | 96.21 |
|  | DBM-Df-1-4 | 2,232,855,278 | 22,107,478 | 51.57 | 98.70 | 96.22 |
| DBM-5 (five days after P) | DBM-Df-5-1 | 1,966,439,296 | 19,469,696 | 53.08 | 98.24 | 94.67 |
|  | DBM-Df-5-2 | 2,680,504,852 | 26,539,652 | 52.80 | 98.81 | 96.39 |
|  | DBM-Df-5-3 | 1,935,046,274 | 19,158,874 | 53.06 | 98.29 | 94.84 |
| **RNAseq trimmed data** | | | | | | |
| Df ovary | Df_ovary-1 | 2,846,913,403 | 28,485,280 | 52.66 | 98.12 | 96.49 |
|  | Df_ovary-2 | 2,623,751,179 | 26,274,390 | 53.30 | 97.81 | 95.87 |
|  | Df_ovary-3 | 2,936,151,249 | 29,493,568 | 52.63 | 97.80 | 95.86 |
| DBM-1 (without P) | DBM-1-2 | 2,787,683,645 | 27,823,240 | 51.10 | 99.18 | 97.07 |
|  | DBM-1-3 | 2,142,396,249 | 21,380,478 | 51.69 | 99.18 | 97.07 |
|  | DBM-1-4 | 2,157,582,003 | 21,535,556 | 51.42 | 99.16 | 96.99 |
| DBM-5 (without P) | DBM-5-1 | 1,989,890,207 | 19,865,276 | 53.49 | 99.24 | 97.22 |
|  | DBM-5-2 | 2,364,101,904 | 23,589,100 | 54.88 | 99.21 | 97.11 |
|  | DBM-5-3 | 1,846,279,631 | 18,422,846 | 52.66 | 99.20 | 97.14 |
| DBM-Df1 (one day after P) | DBM-Df-1-1 | 2,379,390,880 | 23,765,382 | 50.84 | 99.11 | 96.86 |
|  | DBM-Df-1-3 | 2,135,107,796 | 21,321,506 | 52.16 | 99.17 | 97.00 |
|  | DBM-Df-1-4 | 2,181,109,442 | 21,781,470 | 51.61 | 99.17 | 97.04 |
| DBM-Df5 (five days after P) | DBM-Df-5-1 | 1,918,814,037 | 19,211,082 | 53.09 | 98.81 | 95.62 |
|  | DBM-Df-5-2 | 2,631,089,641 | 26,237,274 | 52.81 | 99.19 | 97.06 |
|  | DBM-Df-5-3 | 1,888,121,903 | 18,911,974 | 53.07 | 98.85 | 95.79 |

^#^Df ovary indicates raw and trimmed data of the RNA sequence in the ovary tissue of *Diadegma fenestralae*;

*DBM-1 (without P) indicates the diamond back moth (*Platulla xylostella*) host without parasitization after one day;

** DBM-5 (without P) indicates the diamond back moth (*P. xylostella*) host with or without Parasitization after five days;

^a^Q20% (the percentage quality score of 20) represents an error rate of 1 in 100 (1bp error read in each 100 bp sequencing, call accuracy of 99%), with a corresponding;

^b^Q30% indicates virtually reads will be perfect with no ambiguities.
