# Supplementary material for: Genome, host genome integration, and gene expression in Diadegma fenestrale ichnovirus from the perspective of coevolutionary hosts
Source: Front Microbiol. 2023 Feb 17;14:1035669. doi: 10.3389/fmicb.2023.1035669 (PMC9981800; doi:10.3389/fmicb.2023.1035669)
Supplement: Supplementary file 2 [file Table_7.DOCX]

Supplementary Material

Genome, Host Genome Integration, and Gene Expression in Diadegma fenestrale Ichnovirus from the Perspective of Coevolutionary Hosts

# Juil Kim ^1, 2*,^ Md-Mafizur Rahman^3^, A-Young Kim^4^, Ramasamy Srinivasan^5^, Min Kwon^6^, Yonggyun Kim

*** Correspondence:** Corresponding Author: forweek@kangwon.ac.kr

# Supplementary Figures and Tables

**Supplementary Table 2**. Summarized results of *Diadegma fenestrale* ichnovirus (DfIV) genome sequencing

| Data category | Illumina based DfIV genome sequence | | | | | |
| --- | --- | --- | --- | --- | --- | --- |
|  | Total Bases | | Read Count | GC (%) | ^a^Q20 (%) | ^b^Q30 (%) |
| Raw data | 2,773,322,034 | | 27,458,634 | 44.11 | 99.45 | 95.99 |
| Trimmed & filtered data | 282,800,000 | | 2,800,000 | 44.11 | 99.45 | 95.99 |
| Sequencing methods | DfIV genome assembly results | | | | | |
|  | number of segments | total bases | N50 | longest base | shortest base | average bases |
| 454 based primary genome segments | 65 | 249,541 | 4,462 | 6,685 | 1,444 | 3,839 |
| Hybrid (454 and illumina) | 62 | 247,241 | 4,448 | 8,192 | 1,511 | 3,987 |

^a^Q20% (the percentage quality score of 20) represents an error rate of 1 in 100 (1bp error read in each 100 bp sequencing, call accuracy of 99%), with a corresponding;

^b^Q30% indicates virtually reads will be perfect with no ambiguities.
