# Supplementary material for: Genome, host genome integration, and gene expression in Diadegma fenestrale ichnovirus from the perspective of coevolutionary hosts
Source: Front Microbiol. 2023 Feb 17;14:1035669. doi: 10.3389/fmicb.2023.1035669 (PMC9981800; doi:10.3389/fmicb.2023.1035669)
Supplement: Supplementary file 3 [file Table_8.DOCX]

Supplementary Material

Genome, Host Genome Integration, and Gene Expression in Diadegma fenestrale Ichnovirus from the Perspective of Coevolutionary Hosts

# Juil Kim ^1, 2*,^ Md-Mafizur Rahman^3^, A-Young Kim^4^, Ramasamy Srinivasan^5^, Min Kwon^6^, Yonggyun Kim

*** Correspondence:** Corresponding Author: forweek@kangwon.ac.kr

# Supplementary Figures and Tables

**Supplementary Table 3**. Re-sequencing results for host genome integration motif identification

| Sample | ^a^ Reference length | ^b^ Mapped Sites  (% of over 1 site) | ^c^ Total Reads | ^d^ Mapped Reads  (% of over 1 read) | ^e^ Mean Depth |
| --- | --- | --- | --- | --- | --- |
| DBM-Df-1 | 393,470,562 | 295,564,546 (75.12%) | 119,450,612 | 105,129,279 (88.01%) | 23.93 |
| DBM-Df-3 | 393,470,562 | 295,784,182 (75.17%) | 122,991,414 | 108,278,244 (88.04%) | 24.64 |
| DBM-Df-7 | 393,470,562 | 294,783,131 (74.92%) | 120,979,494 | 106,195,781 (87.78%) | 24.17 |

^a^ Reference length: Length of reference genome (lepidopteran host, diamond back moth, *Platulla xylostella* GCA_000330985.1);

^b^ Mapped Sites: total length of mapped site;

^c^ Total Reads: Number of total read;

^d^ Mapped Reads: Number of reads mapped to the reference;

^e^ Mean Depth: Average alignment depth
