# Supplementary material for: Genome, host genome integration, and gene expression in Diadegma fenestrale ichnovirus from the perspective of coevolutionary hosts
Source: Front Microbiol. 2023 Feb 17;14:1035669. doi: 10.3389/fmicb.2023.1035669 (PMC9981800; doi:10.3389/fmicb.2023.1035669)
Supplement: Supplementary file 4 [file Table_9.DOCX]

Supplementary Material

Genome, Host Genome Integration, and Gene Expression in Diadegma fenestrale Ichnovirus from the Perspective of Coevolutionary Hosts

# Juil Kim ^1, 2*,^ Md-Mafizur Rahman^3^, A-Young Kim^4^, Ramasamy Srinivasan^5^, Min Kwon^6^, Yonggyun Kim

*** Correspondence:** Corresponding Author: forweek@kangwon.ac.kr

**Supplementary Table 4**. Pictorial representation of host genome integration motifs, their inserted genes, chromosome number and segments, read depth, and sequence motifs with three biological replications: i). DBM-Df-1, ii). DBM-Df-3, and iii). DBM-Df-7)

1. DBM-Df-1

| Inserted Gene | chr ^*^ | Genomic segments | Read Depth^&^ | Motif Length (bp) | Motif Sequence on DBM | Motif Sequence on DfIV (Insertion site 1) | | DBM Sequence | | Motif Sequence on DfIV (Insertion site 2) | | Motif Sequence on DBM |
| --- | --- | --- | --- | --- | --- | --- | --- | --- | --- | --- | --- | --- |
|  |  |  |  |  |  |  |  | Start Position | End Position |  |  |  |
| **DfIV_F2_6602** | NW_011952513.1 | **F2-1** | 24 | 10 | CACTTGCAAT | ATTAATTGTAATGTATGTCGCCTTATTATGTGATA | | ATAATAATAATAATAATAATAAATATATTT | | TTGCACATATGTGTATGGCTATTTCTTTCTAATGTA | | ATTTTCAGACC |
|  |  |  |  | 20 | TGCCGCCCAACACTTGCAAT |  |  |  |  |  |  | ATTTTCAGACCTCATAGTCCA |
|  |  |  |  | 50 | CAGCACTAGACGTAGGACTAGCGGCGCCACTGCCGCCCAACACTTGCAAT |  |  |  |  |  |  | ATTTTCAGACCTCATAGTCCATAGTAAGTGTTAGTAACAATGAATCTTAAT |
|  |  |  |  |  |  | 1847 | 1881 | 5040 | 5069 | 2032 | 2067 |  |
|  | NW_011952013.1 | **F2-2** | 21 | 10 | ACCCCACATA | GCCATACACATATGTGCAAAAATATAT | | TTATTATTATTATTATTATTATTATCACAT | | AATAAGGCGACATACATTACAATTAATTCTAGGTTGAAGTAAAT | | GATCGTGGTC |
|  |  |  |  | 20 | GTGTGAGATGACCCCACATA |  |  |  |  |  |  | GATCGTGGTCGAAAACAATC |
|  |  |  |  | 50 | ATTCTGCCTAGCTTGGGGTCGGATGGCCGTGTGTGAGATGACCCCACATA |  |  |  |  |  |  | GATCGTGGTCGAAAACAATCGGCGACAGATTGTAGAGTTCCCGCCTAGCT |
|  |  |  |  |  |  | 1930 | 1904 | 443491 | 443520 | 1873 | 1830 |  |
|  | NW_011952577.1 | **F2-3** | 16 | 10 | AATCGCACAG | TACATTAGAAAGAAATAGCCATACACATATGTGCAAAAA | | TATATTTATTATTATTATTATTATTATTAT | | CACATAATAAGGCGACATACATTACAATTAAT | | TTAACCTTTTA |
|  |  |  |  | 20 | TTTATCTAACAATCGCACAG |  |  |  |  |  |  | TTAACCTTTTACTGTACTGGT |
|  |  |  |  | 50 | CACAAATACAATATGGAATTGATCTATATCTTTATCTAACAATCGCACAG |  |  |  |  |  |  | TTAACCTTTTACTGTACTGGTATTATTTTTAATCATATTATAGTTGGTTTT |
|  |  |  |  |  |  | 1947 | 1909 | 102877 | 102906 | 1878 | 1847 |  |
|  | NW_011952122.1 | **F2-4** | 12 | 10 | TATCAAATAA | TACATTAGAAAGAAATAGCCATACACATATGTGCAAAAAT | | ATATTTATTATTATTATTATTATTATTATCACATA | | ATAAGGCGACATACATTACAATTAAT | | TAAATCTGTG |
|  |  |  |  | 20 | CTAAACTGTTTATCAAATAA |  |  |  |  |  |  | TAAATCTGTGTACAAACGTA |
|  |  |  |  | 50 | ATCAACTAGTTAAGAATTTTGTGTAACAAACTAAACTGTTTATCAAATAA |  |  |  |  |  |  | TAAATCTGTGTACAAACGTAGTTCGTTTTTATATTATTTAAACGGAGTTT |
|  |  |  |  |  |  | 1947 | 1908 | 583817 | 583851 | 1872 | 1847 |  |
|  | NW_011952193.1 | **F2-5** | 15 | 10 | TAATGTTTTA | AATGTATGTCGCCTTATTATGT | | GATAATAATAATAATAATAATAATAATAAATAT | | ATTTTTGCACATATGTGTATGGCTATTTCTTTCTAATGTTACCTTA | | GTAGGGACAT |
|  |  |  |  | 20 | CATCTAAAACTAATGTTTTA |  |  |  |  |  |  | GTAGGGACATCTCACACACG |
|  |  |  |  | 50 | TACTAAATACAAGTGTTTTGTGACTAAAACCATCTAAAACTAATGTTTTA |  |  |  |  |  |  | GTAGGGACATCTCACACACGGCCATCCAACCCCAAGCTAGGCAGAGCCTG |
|  |  |  |  |  |  | 1856 | 1877 | 17071 | 17103 | 1908 | 1946 |  |
| **DfIV_E3_5528** | NW_011952077.1 | **E3** | 29 | 10 | GTTTATAATT | TGATGCGAAGTGATCATATACACACACTCCG | | ATACTACTACTACTACTACTACTCCTACCACTACTACTATTACTAT | | AGTTTACAACGTTGTAGTGACAGC | | TATAGGTATT |
|  |  |  |  | 20 | AACAAGAAAAGTTTATAATT |  |  |  |  |  |  | TATAGGTATTTTTTGTACTT |
|  |  |  |  | 50 | ATTTTTTGAGCGGGTGCATATTTTTTATGAAACAAGAAAAGTTTATAATT |  |  |  |  |  |  | TATAGGTATTTTTTGTACTTACGTGTGCATATTAACATTAATTGTATCGA |
|  |  |  |  |  |  | 2657 | 2627 | 26066 | 26111 | 2580 | 2557 |  |
| **DfIV_D22_4930** | NW_011952077.1 | **D22** | 15 | 10 | GTTTATAATT | ACCATGATGCGAAGTGATTATACATAC | | ATACTACTACTACTACTACTACTACTAATACTACT | | GCCACTTCTATTACTATATCGCTATATGCTCACGGGCCATGGCCTCTTC | | ACTACTACTA |
|  |  |  |  | 20 | AACAAGAAAAGTTTATAATT |  |  |  |  |  |  | ACTACTACTATTATAGGTAT |
|  |  |  |  | 50 | ATTTTTTGAGCGGGTGCATATTTTTTATGAAACAAGAAAAGTTTATAATT |  |  |  |  |  |  | ACTACTACTATTATAGGTATTTTTTGTACTTACGTGTGCATATTAACATT |
|  |  |  |  |  |  | 2616 | 2593 | 26066 | 26100 | 2518 | 2470 |  |

*means chromosome; **means read depth ≥ 15 bp inserted Genome

1. DBM-Df-3

| Inserted Gene | chr * | Read Depth** | Motif Length (bp) | Motif Sequence on DBM | Motif Sequence on DfIV (Insertion site 1) | | DBM Sequence | | Motif Sequence on DfIV (Insertion site 2) | | Motif Sequence on DBM |
| --- | --- | --- | --- | --- | --- | --- | --- | --- | --- | --- | --- |
|  |  |  |  |  |  |  | Start Position | End Position |  |  |  |
| DfIV_F2_6602 | NW_011952513.1 | 19 | 10 | CACTTGCAAT | ATTAATTGTAATGTATGTCGCCTTATTATGTGATA | | ATAATAATAATAATAATAATAAATATATTT | | TTGCACATATGTGTATGGCTATTTCTTTCTAATGTA | | ATTTTCAGACC |
|  |  |  | 20 | TGCCGCCCAACACTTGCAAT |  |  |  |  |  |  | ATTTTCAGACCTCATAGTCCA |
|  |  |  | 50 | CAGCACTAGACGTAGGACTAGCGGCGCCACTGCCGCCCAACACTTGCAAT |  |  |  |  |  |  | ATTTTCAGACCTCATAGTCCATAGTAAGTGTTAGTAACAATGAATCTTAAT |
|  |  |  |  |  | 1847 | 1881 | 5040 | 5069 | 2032 | 2067 |  |
|  | NW_011952013.1 | 19 | 10 | ACCCCACATA | GCCATACACATATGTGCAAAAATATAT | | TTATTATTATTATTATTATTATTATCACAT | | AATAAGGCGACATACATTACAATTAATTCTAGGTTGAAGTAAAT | | GATCGTGGTC |
|  |  |  | 20 | GTGTGAGATGACCCCACATA |  |  |  |  |  |  | GATCGTGGTCGAAAACAATC |
|  |  |  | 50 | ATTCTGCCTAGCTTGGGGTCGGATGGCCGTGTGTGAGATGACCCCACATA |  |  |  |  |  |  | GATCGTGGTCGAAAACAATCGGCGACAGATTGTAGAGTTCCCGCCTAGCT |
|  |  |  |  |  | 1930 | 1904 | 443491 | 443520 | 1873 | 1830 |  |
|  | NW_011952577.1 | 20 | 10 | AATCGCACAG | TACATTAGAAAGAAATAGCCATACACATATGTGCAAAAA | | TATATTTATTATTATTATTATTATTATTAT | | CACATAATAAGGCGACATACATTACAATTAAT | | TTAACCTTTTA |
|  |  |  | 20 | TTTATCTAACAATCGCACAG |  |  |  |  |  |  | TTAACCTTTTACTGTACTGGT |
|  |  |  | 50 | CACAAATACAATATGGAATTGATCTATATCTTTATCTAACAATCGCACAG |  |  |  |  |  |  | TTAACCTTTTACTGTACTGGTATTATTTTTAATCATATTATAGTTGGTTTT |
|  |  |  |  |  | 1947 | 1909 | 102877 | 102906 | 1878 | 1847 |  |
|  | NW_011952122.1 | 15 | 10 | TATCAAATAA | TACATTAGAAAGAAATAGCCATACACATATGTGCAAAAAT | | ATATTTATTATTATTATTATTATTATTATCACATA | | ATAAGGCGACATACATTACAATTAAT | | TAAATCTGTG |
|  |  |  | 20 | CTAAACTGTTTATCAAATAA |  |  |  |  |  |  | TAAATCTGTGTACAAACGTA |
|  |  |  | 50 | ATCAACTAGTTAAGAATTTTGTGTAACAAACTAAACTGTTTATCAAATAA |  |  |  |  |  |  | TAAATCTGTGTACAAACGTAGTTCGTTTTTATATTATTTAAACGGAGTTT |
|  |  |  |  |  | 1947 | 1908 | 583817 | 583851 | 1872 | 1847 |  |
|  | NW_011952193.1 | 9 | 10 | TAATGTTTTA | AATGTATGTCGCCTTATTATGT | | GATAATAATAATAATAATAATAATAATAAATAT | | ATTTTTGCACATATGTGTATGGCTATTTCTTTCTAATGTTACCTTA | | GTAGGGACAT |
|  |  |  | 20 | CATCTAAAACTAATGTTTTA |  |  |  |  |  |  | GTAGGGACATCTCACACACG |
|  |  |  | 50 | TACTAAATACAAGTGTTTTGTGACTAAAACCATCTAAAACTAATGTTTTAGA |  |  |  |  |  |  | GTAGGGACATCTCACACACGGCCATCCAACCCCAAGCTAGGCAGAGCCTG |
|  |  |  |  |  | 1856 | 1877 | 17071 | 17103 | 1908 | 1946 |  |
| DfIV_E3_5528 | NW_011952077.1 | 26 | 10 | GTTTATAATT | TGATGCGAAGTGATCATATACACACACTCCG | | ATACTACTACTACTACTACTACTCCTACCACTACTACTATTACTAT | | AGTTTACAACGTTGTAGTGACAGC | | TATAGGTATT |
|  |  |  | 20 | AACAAGAAAAGTTTATAATT |  |  |  |  |  |  | TATAGGTATTTTTTGTACTT |
|  |  |  | 50 | ATTTTTTGAGCGGGTGCATATTTTTTATGAAACAAGAAAAGTTTATAATT |  |  |  |  |  |  | TATAGGTATTTTTTGTACTTACGTGTGCATATTAACATTAATTGTATCGA |
|  |  |  |  |  | 2657 | 2627 | 26066 | 26111 | 2580 | 2557 |  |
| DfIV_D22_4930 | NW_011952077.1 | 24 | 10 | GTTTATAATT | ACCATGATGCGAAGTGATTATACATAC | | ATACTACTACTACTACTACTACTACTAATACTACT | | GCCACTTCTATTACTATATCGCTATATGCTCACGGGCCATGGCCTCTTC | | ACTACTACTA |
|  |  |  | 20 | AACAAGAAAAGTTTATAATT |  |  |  |  |  |  | ACTACTACTATTATAGGTAT |
|  |  |  | 50 | ATTTTTTGAGCGGGTGCATATTTTTTATGAAACAAGAAAAGTTTATAATT |  |  |  |  |  |  | ACTACTACTATTATAGGTATTTTTTGTACTTACGTGTGCATATTAACATT |
|  |  |  |  |  | 2616 | 2593 | 26066 | 26100 | 2518 | 2470 |  |

*means chromosome; **means read depth ≥ 15 bp inserted Genome

1. DBM-Df-7

| Inserted Gene | chr* | Read Depth** | Motif Length (bp) | Motif Sequence on DBM | Motif Sequence on DfIV (Insertion site 1) | | DBM Sequence | | Motif Sequence on DfIV (Insertion site 2) | | Motif Sequence on DBM |
| --- | --- | --- | --- | --- | --- | --- | --- | --- | --- | --- | --- |
|  |  |  |  |  |  |  | Start Position | End Position |  |  |  |
| DfIV_F2_6602 | NW_011952513.1 | 51 | 10 | CACTTGCAAT | ATTAATTGTAATGTATGTCGCCTTATTATGTGATA | | ATAATAATAATAATAATAATAAATATATTT | | TTGCACATATGTGTATGGCTATTTCTTTCTAATGTA | | ATTTTCAGACC |
|  |  |  | 20 | TGCCGCCCAACACTTGCAAT |  |  |  |  |  |  | ATTTTCAGACCTCATAGTCCA |
|  |  |  | 50 | CAGCACTAGACGTAGGACTAGCGGCGCCACTGCCGCCCAACACTTGCAAT |  |  |  |  |  |  | ATTTTCAGACCTCATAGTCCATAGTAAGTGTTAGTAACAATGAATCTTAAT |
|  |  |  |  |  | 1847 | 1881 | 5040 | 5069 | 2032 | 2067 |  |
|  | NW_011952013.1 | 35 | 10 | ACCCCACATA | GCCATACACATATGTGCAAAAATATAT | | TTATTATTATTATTATTATTATTATCACAT | | AATAAGGCGACATACATTACAATTAATTCTAGGTTGAAGTAAAT | | GATCGTGGTC |
|  |  |  | 20 | GTGTGAGATGACCCCACATA |  |  |  |  |  |  | GATCGTGGTCGAAAACAATC |
|  |  |  | 50 | ATTCTGCCTAGCTTGGGGTCGGATGGCCGTGTGTGAGATGACCCCACATA |  |  |  |  |  |  | GATCGTGGTCGAAAACAATCGGCGACAGATTGTAGAGTTCCCGCCTAGCT |
|  |  |  |  |  | 1930 | 1904 | 443491 | 443420 | 1873 | 1830 |  |
|  | NW_011952577.1 | 35 | 10 | AATCGCACAG | TACATTAGAAAGAAATAGCCATACACATATGTGCAAAAA | | TATATTTATTATTATTATTATTATTATTAT | | CACATAATAAGGCGACATACATTACAATTAAT | | TTAACCTTTTA |
|  |  |  | 20 | TTTATCTAACAATCGCACAG |  |  |  |  |  |  | TTAACCTTTTACTGTACTGGT |
|  |  |  | 50 | CACAAATACAATATGGAATTGATCTATATCTTTATCTAACAATCGCACAG |  |  |  |  |  |  | TTAACCTTTTACTGTACTGGTATTATTTTTAATCATATTATAGTTGGTTTT |
|  |  |  |  |  | 1947 | 1909 | 102877 | 102906 | 1878 | 1847 |  |
|  | NW_011952122.1 | 36 | 10 | TATCAAATAA | TACATTAGAAAGAAATAGCCATACACATATGTGCAAAAAT | | ATATTTATTATTATTATTATTATTATTATCACATA | | ATAAGGCGACATACATTACAATTAAT | | TAAATCTGTG |
|  |  |  | 20 | CTAAACTGTTTATCAAATAA |  |  |  |  |  |  | TAAATCTGTGTACAAACGTA |
|  |  |  | 50 | ATCAACTAGTTAAGAATTTTGTGTAACAAACTAAACTGTTTATCAAATAA |  |  |  |  |  |  | TAAATCTGTGTACAAACGTAGTTCGTTTTTATATTATTTAAACGGAGTTT |
|  |  |  |  |  | 1947 | 1908 | 583817 | 583851 | 1872 | 1847 |  |
| DfIV_E3_5528 | NW_011952077.1 | 41 | 10 | GTTTATAATT | TGATGCGAAGTGATCATATACACACACTCCG | | ATACTACTACTACTACTACTACTCCTACCACTACTACTATTACTAT | | AGTTTACAACGTTGTAGTGACAGC | | TATAGGTATT |
|  |  |  | 20 | AACAAGAAAAGTTTATAATT |  |  |  |  |  |  | TATAGGTATTTTTTGTACTT |
|  |  |  | 50 | ATTTTTTGAGCGGGTGCATATTTTTTATGAAACAAGAAAAGTTTATAATT |  |  |  |  |  |  | TATAGGTATTTTTTGTACTTACGTGTGCATATTAACATTAATTGTATCGA |
|  |  |  |  |  | 2657 | 2627 | 26066 | 26111 | 2580 | 2557 |  |
| DfIV_D22_4930 | NW_011952077.1 | 26 | 10 | GTTTATAATT | ACCATGATGCGAAGTGATTATACATAC | | ATACTACTACTACTACTACTACTACTAATACTACTGCCACTTCTATTA | | GCCACTTCTATTACTATATCGCTATATGCTCACGGGCCATGGCCTCTTC | | ACTACTACTA |
|  |  |  | 20 | AACAAGAAAAGTTTATAATT |  |  |  |  |  |  | ACTACTACTATTATAGGTAT |
|  |  |  | 50 | ATTTTTTGAGCGGGTGCATATTTTTTATGAAACAAGAAAAGTTTATAATT |  |  |  |  |  |  | ACTACTACTATTATAGGTATTTTTTGTACTTACGTGTGCATATTAACATT |
|  |  |  |  |  | 2616 | 2593 | 26066 | 26100 | 2518 | 2470 |  |

*means chromosome; **means read depth ≥ 15 bp inserted Genome
