# Supplementary material for: Genome, host genome integration, and gene expression in Diadegma fenestrale ichnovirus from the perspective of coevolutionary hosts
Source: Front Microbiol. 2023 Feb 17;14:1035669. doi: 10.3389/fmicb.2023.1035669 (PMC9981800; doi:10.3389/fmicb.2023.1035669)
Supplement: Supplementary file 5 [file Image_1.pdf]

Supplementary Material

Genome, Host Genome Integration, and Gene Expression in Diadegma fenestrale Ichnovirus from the Perspective of Coevolutionary Hosts

Juil Kim [1, 2*,] Md-Mafizur Rahman[3], A-Young Kim[4], Ramasamy Srinivasan[5], Min Kwon[6], Yonggyun Kim

*** Correspondence:** Corresponding Author: forweek@kangwon.ac.kr

1 Supplementary Figures and Tables

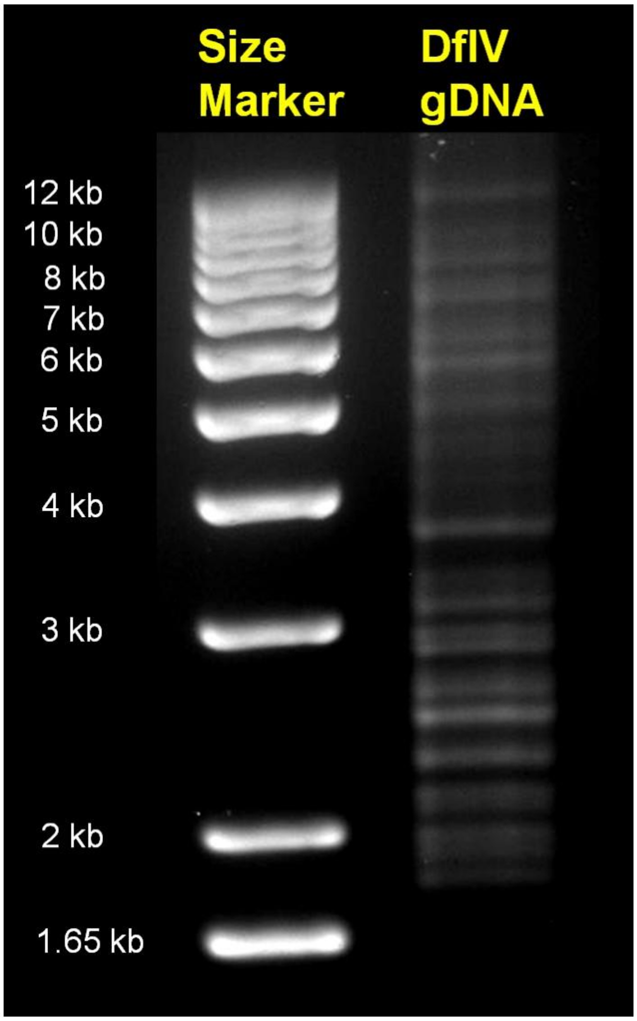

Supplementary Figure 1. Genome segment structure was visualized using EtBr staining following gel electrophoresis. DfIV gDNA (2 µg) was separated onto 0.5% agarose gel at 30 V for 9 h.
