# Supplementary material for: Genome, host genome integration, and gene expression in Diadegma fenestrale ichnovirus from the perspective of coevolutionary hosts
Source: Front Microbiol. 2023 Feb 17;14:1035669. doi: 10.3389/fmicb.2023.1035669 (PMC9981800; doi:10.3389/fmicb.2023.1035669)
Supplement: Supplementary file 6 [file Image_2.pdf]

Supplementary Material

Genome, Host Genome Integration, and Gene Expression in Diadegma fenestrale Ichnovirus from the Perspective of Coevolutionary Hosts

Juil Kim [1, 2*,] Md-Mafizur Rahman[3], A-Young Kim[4], Ramasamy Srinivasan[5], Min Kwon[6], Yonggyun Kim

*** Correspondence:** Corresponding Author: forweek@kangwon.ac.kr

1 Supplementary Figures and Tables

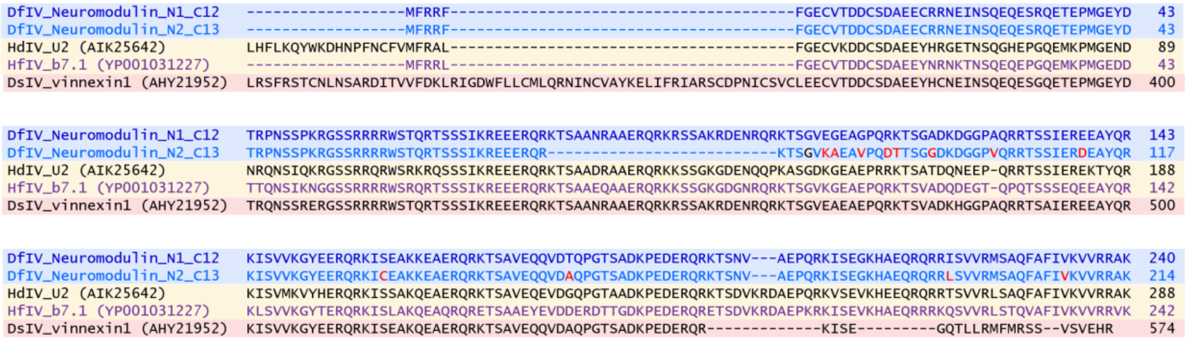

Supplementary Figure 2. Alignment of two gene sequences of *neuromodulin N* with other closely related three sequences (DfIV_Neuromodulin_N1_C12, DfIV_Neuromodulin_N2_C13, HdIV_U2 [AIK25642], HfIV_b7.1 [YP001031227], DsIV_vinnexin1 [AHY21952], wherein we observed a deletion mutation on the aligned sequence. The red-highlighted amino acids indicate differences between DfIV neuromodulins N1 and N2.
