# Supplementary material for: Genome, host genome integration, and gene expression in Diadegma fenestrale ichnovirus from the perspective of coevolutionary hosts
Source: Front Microbiol. 2023 Feb 17;14:1035669. doi: 10.3389/fmicb.2023.1035669 (PMC9981800; doi:10.3389/fmicb.2023.1035669)
Supplement: Supplementary file 7 [file Image_3.pdf]

Supplementary Material

Genome, Host Genome Integration, and Gene Expression in Diadegma fenestrale Ichnovirus from the Perspective of Coevolutionary Hosts

Juil Kim [1, 2*,] Md-Mafizur Rahman[3], A-Young Kim[4], Ramasamy Srinivasan[5], Min Kwon[6], Yonggyun Kim

*** Correspondence:** Corresponding Author: forweek@kangwon.ac.kr

1 Supplementary Figures and Tables

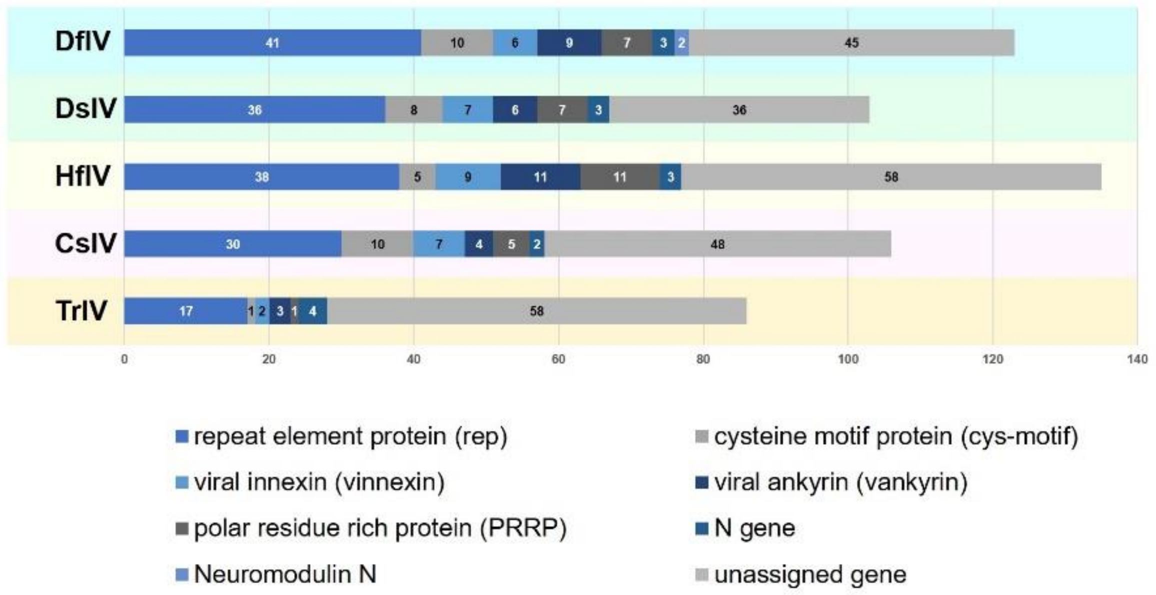

Supplementary Figure 3. Comparative features of DfIV and DsIV ORFs, predicted using ORF finder. A total of 123 and 108 genes were annotated, respectively. Six main ichnovirus (IV) gene families (*rep*, *cys*, *vinnexin*, *vankyrin*, *PRRP*, and *N* genes) and unassigned genes were compared with those of other identified IVs, including TrIV (Tanaka et al., 2007), CsIV (Webb et al., 2006), and HfIV (Tanaka et al., 2007).
