# Supplementary material for: Genome, host genome integration, and gene expression in Diadegma fenestrale ichnovirus from the perspective of coevolutionary hosts
Source: Front Microbiol. 2023 Feb 17;14:1035669. doi: 10.3389/fmicb.2023.1035669 (PMC9981800; doi:10.3389/fmicb.2023.1035669)
Supplement: Supplementary file 8 [file Image_4.pdf]

Supplementary Material

Genome, Host Genome Integration, and Gene Expression in Diadegma fenestrale Ichnovirus from the Perspective of Coevolutionary Hosts

Juil Kim [1, 2*], Md-Mafizur Rahman[3], A-Young Kim[4], Ramasamy Srinivasan[5], Min Kwon[6], Yonggyun Kim

*** Correspondence:** Corresponding Author: forweek@kangwon.ac.kr

1 Supplementary Figures and Tables

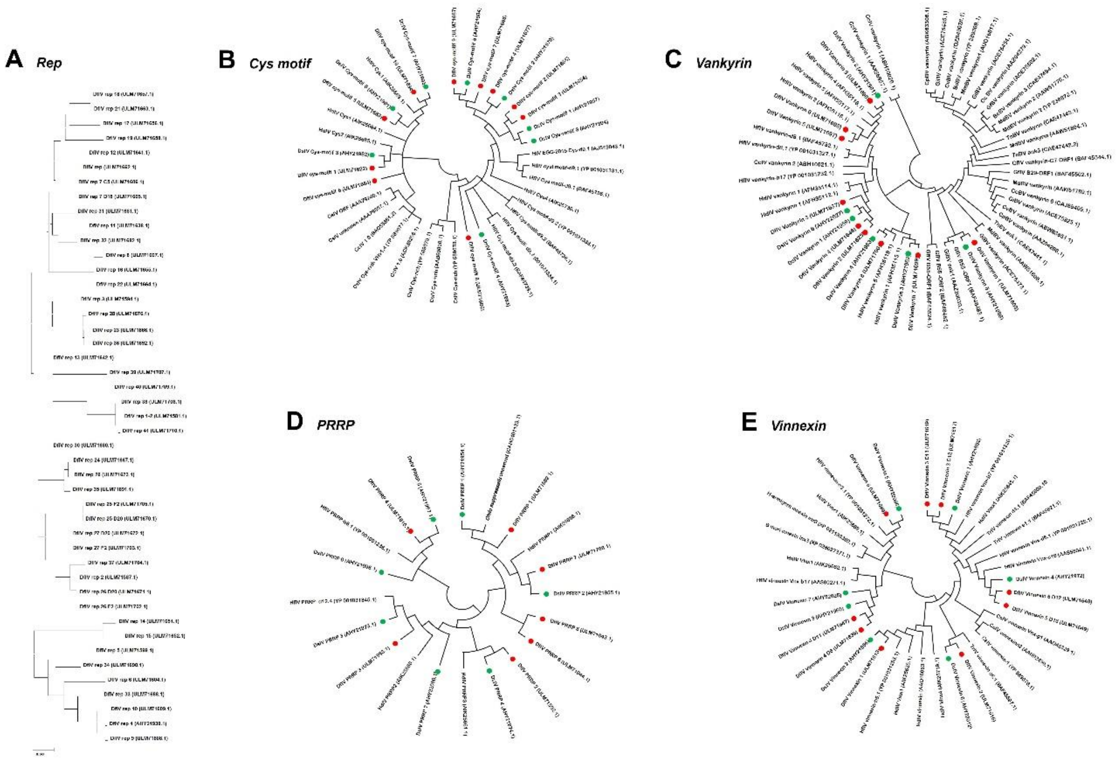

Supplementary Figure 4 (A-E). **A).** Phylogenetic analysis of repeat element proteins (Rep). An unrooted JTT+G model was used for Mega cladogram construction of rep proteins. One DfIV protein was clearly smaller than the others and was not included in this analysis. Forty-one (n = 41) rep proteins were used for phylogenetic analysis. **B).** Cys-motif of DfIV and DsIV were combined with NCBI-acquired cys-motif protein sequences for phylogenetic analysis. **C).** DfIV and DsIV Vankyrins proteins were combined with NCBI-acquired Vankyrin sequences for the construction of a phylogenetic tree. **D).** PRRP proteins of DfIV and DsIV were combined with NCBI-acquired genes for construction of a phylogenetic tree. **E).** The Vinnexin proteins were used for construction of

phylogenetic trees and the subsequent analysis. The red and green colors indicate DfIV and DsIV protein codes, respectively. The protein codes and parenthesis numbers are shown in closed brackets.
