# Supplementary material for: Genome, host genome integration, and gene expression in Diadegma fenestrale ichnovirus from the perspective of coevolutionary hosts
Source: Front Microbiol. 2023 Feb 17;14:1035669. doi: 10.3389/fmicb.2023.1035669 (PMC9981800; doi:10.3389/fmicb.2023.1035669)
Supplement: Supplementary file 9 [file Image_5.pdf]

Supplementary Material

Genome, Host Genome Integration, and Gene Expression in Diadegma fenestrale Ichnovirus from the Perspective of Coevolutionary Hosts

Juil Kim [1, 2*,] Md-Mafizur Rahman[3], A-Young Kim[4], Ramasamy Srinivasan[5], Min Kwon[6], Yonggyun Kim

*** Correspondence:** Corresponding Author: forweek@kangwon.ac.kr

1 Supplementary Figures and Tables

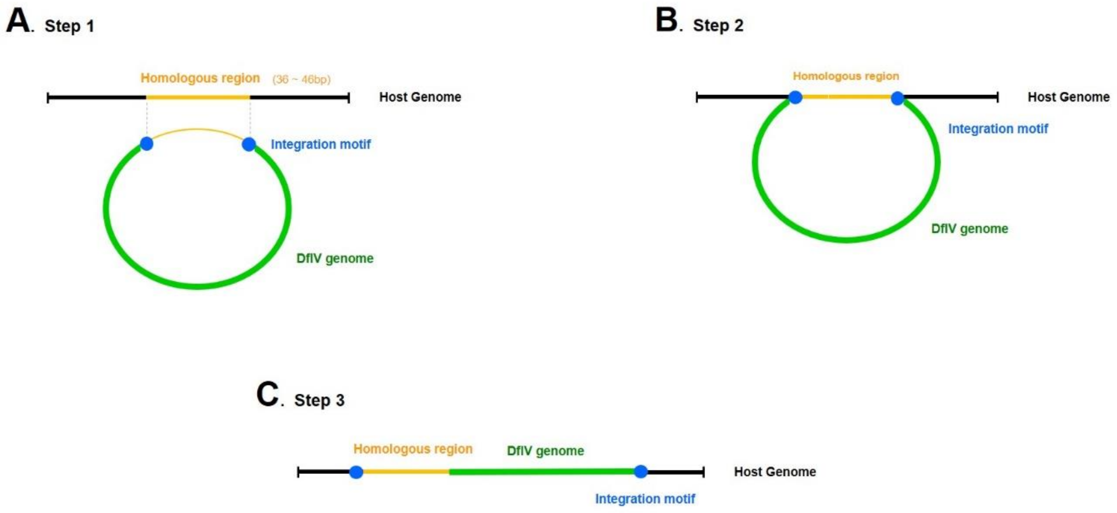

Supplementary Figure 5. A proposed schematic diagram of the integration mechanism between *Diadegma fenestrale* ichnovirus (DfIV) and the diamondback moth (*Plutella xylostella*) genome. **A).** The homologous sequences ranged in length from 36–46 bp between circular viral genomic (DfIV) segments and host genome segments, and integration motifs flanked homologous regions. **B).** Host and viral homologous regions were integrated and surrounded by the flanking sites of the integration motif. **C).** The homologous region and integrated motifs were combined into a partial linearized host genome during the homologous recombination process (Legeai et al., 2020). Detailed information (re-sequencing results, integration motif sequences and their positions in the host genome, homologous sequences and their positions) is provided in supplementary Table S4 and Figure 2.
